# Versatile Liquid Metal Composite Inks for Printable, Durable, and Ultra‐Stretchable Electronics

**DOI:** 10.1002/smll.202501829

**Published:** 2025-06-11

**Authors:** Jeongsu Pyeon, Hyeonseung Lee, Wonho Choe, Sanghoo Park, Hyoungsoo Kim

**Affiliations:** ^1^ Department of Mechanical Engineering Korea Advanced Institute of Science and Technology Daejeon 34141 Republic of Korea; ^2^ Department of Nuclear and Quantum Engineering Korea Advanced Institute of Science and Technology Daejeon 34141 Republic of Korea

**Keywords:** durability, liquid metal composite ink, metamaterial absorber, printing, self‐sintering, stretchability

## Abstract

Liquid metal (LM) offers exceptional potential for stretchable and flexible applications due to its outstanding stretchability, flexibility, and electrical conductivity. The inherent characteristics of LM–such as high surface tension, low viscosity, poor wettability, and sintering challenges–pose significant obstacles for use in commercial printing. To overcome these, a self‐sintering liquid metal composite particle (LMCP) ink with tunable surface tension, viscosity, and wettability is developed. LMCP inks are tailored for commercial printing processes requiring low surface tension and high viscosity, enabling printing on diverse substrates. The ink achieves coffee‐ring‐free, crack‐free, bilayer‐free, and post‐processing‐free deposition through simple evaporation under ambient conditions, while preserving electrical conductivity. Key innovations include: i) utilizing polyvinylpyrrolidone (PVP)‐capped liquid metal particles (LMPs) to reduce surface tension and enhance wettability; ii) incorporating Laponite as a viscosity enhancer and leveraging LMP sedimentation to control viscosity; iii) forming LMCPs encapsulated by PVP and Laponite through co‐self‐assembly on LMP surfaces to promote self‐sintering; and iv) achieving uniform deposition via solutal‐Marangoni‐driven mixing and particle settling. The resulting LMCP electrodes exhibit over 1200% stretchability, patternability into complex configurations, and stability for nearly one year in air. Additionally, their application as stretchable metamaterial absorbers is highlighted, showcasing their potential in advanced printable electronics.

## Graphical abstract

A printable liquid metal composite ink with self‐sintering capability, high stretchability, and long‐term durability is developed. It enables crack‐free, high‐resolution patterns on flexible substrates using ambient drying. The ink demonstrates potential in stretchable electronics, especially in tunable metamaterial absorbers, offering a scalable and robust platform for next‐generation soft electronic applications.

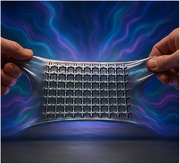

## 1 Introduction

In recent years, room‐temperature liquid metal (LM) has been highlighted as a promising conductive material for next‐generation stretchable and flexible electronics, including soft sensors and actuators,^[^ ^1^ ^]^ wearable electronic skins,^[^ ^2^ ^]^ stretchable displays,^[^ ^3^ ^]^ and batteries.^[^ ^4^ ^]^ This increasing interest is primarily due to its high electrical conductivity, even under extreme stretching conditions, and its inherent flexibility (see Table S1, Supporting Information) as well as its low toxicity compared to mercury. These unique characteristics of LMs facilitate the straightforward printing of conductive electrode patterns on a wide range of surfaces. Among various types of LM, gallium‐based alloys, especially Galinstan (GaInSn), have drawn significant attention due to their ability to function effectively at sub‐zero temperatures. This is due to its low melting point, while EGaIn loses its fluidic properties at near‐ and sub‐zero temperatures; specifically, the melting temperature of Galinstan (Ga 68.5 wt%, In 21.5 wt%, and Sn 10.0 wt%) is −19.0 °C, whereas that of EGaIn (Ga 75.5 wt%, and In 24.5 wt%) is 15.5 °C.^[^ ^5^ ^]^ However, several critical challenges arise when coating LM electrodes onto target substrates using commercial printing techniques such as inkjet, spray, stencil, roll‐to‐roll, and blade printing. These challenges include: i) the high surface tension of LM (greater than 0.5 N m^−1^ for Galinstan),^[^ ^6^ ^]^ ii) its poor wettability on substrates, and iii) its low viscosity range (approximately 2.4 cP for Galinstan).^[^ ^5^ ^]^

In particular, the high surface tension of liquid metals poses challenges for their delivery through narrow nozzles in dispensing systems, typically ranging from hundreds to tens of micrometers in size. This often results in jetting reliability issues,^[^ ^7^, ^8^ ^]^ necessitating the use of high‐pressure equipment or high‐voltage pulse operations to overcome these difficulties. The poor wettability also limits electrode fabrication on substrates with low surface energy and uneven topographies, such as stretchable and flexible materials.^[^ ^8^, ^9^, ^10^ ^]^ Furthermore, the low viscosity of LMs restricts large‐scale and mass‐production printing, as typical inks require viscosities from at least 10 to 500 cP.^[^ ^11^ ^]^ While gel‐based LM inks have demonstrated impressive conductivities, they are primarily suited for extrusion‐based printing due to their high yield stress and viscoelasticity.^[^ ^12^, ^13^ ^]^ Such systems typically lack the rheological adaptability required for 2D printing techniques such as stencil, doctor blade, or roll‐to‐roll printing. In contrast, our ink formulation is explicitly designed for compatibility with planar and scalable coating processes by simultaneously achieving tunable viscosity, low surface tension, and high substrate wettability.

**Figure 1:**
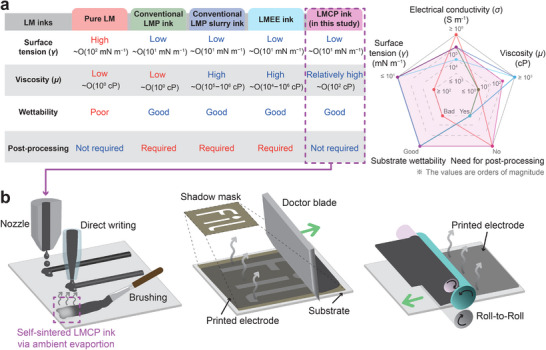
Liquid metal composite particle (LMCP) ink. a) Comparison of the characteristics of printing inks based on liquid metal (LM), including pure LM inks,^[^ ^5^, ^9^, ^10^ ^]^ conventional liquid metal particle (LMP) inks,^[^ ^14^, ^15^, ^16^, ^18^, ^19^ ^]^ conventional LMP slurry inks,^[^ ^21^, ^22^, ^23^ ^]^ liquid metal‐embedded elastomer (LMEE) inks,^[^ ^25^, ^26^, ^27^, ^28^ ^]^ and the current LMCP ink. The viscosities (*µ*) at a shear rate of approximately 1 s^−1^ are compared for each ink. Post‐processing involves sintering, heat‐assisted solvent drying, and elastomer curing. The detailed fabrication process of the LMCP ink is illustrated in Figure S1 (Supporting Information). b) Demonstration of the versatility of LMCP ink with various conventional printing techniques, including inkjet printing, direct writing, brushing, doctor blade coating, and roll‐to‐roll printing (from left to right).

To address challenges in liquid metal (LM) patterning, researchers have developed liquid metal particle (LMP) inks, which reduce surface tension and enhance wettability on various substrates. These improvements are achieved by incorporating low surface tension solvents, such as ethanol (EtOH), acetone, and deionized (DI) water, enabling better ink spreading.^[^ ^14^, ^15^, ^16^, ^17^, ^18^, ^19^ ^]^ However, while LMP inks mitigate coating difficulties, they introduce new challenges, particularly the formation of oxide layers on the LMP surface,^[^ ^14^ ^]^ requiring post‐treatment. To tackle this, methods such as mechanical pressing/stretching,^[^ ^14^, ^15^, ^16^ ^]^ thermal laser/heating treatments,^[^ ^17^, ^18^ ^]^ and acoustic field application^[^ ^20^ ^]^ have been explored to activate the electrical conductivity of LMP electrodes. These additional steps, however, complicate the production process and hinder scalability. Another drawback of conventional LMP inks is their low viscosity, typically below 10 cP, which limits their compatibility with diverse printing techniques. To overcome this, LMP slurry inks have emerged as a promising alternative.^[^ ^21^, ^22^, ^23^, ^24^ ^]^ These inks achieve higher viscosity–ranging from 10^5^ to 10^6^ cP–by increasing LMPs' density through sedimentation. This process is driven either by the large density difference between LMPs (approximately 6440 kg m^−3^ for Galinstan) and the surrounding liquid (typically 784–997 kg m^−3^) or by centrifuge‐assisted sedimentation. Additionally, biphasic LM mixtures, i.e., liquid metal‐embedded elastomer (LMEE) inks with viscosities of 10^4^–10^6^ cP, have been proposed, formulated with highly viscous solvents like polydimethylsiloxane (PDMS) and optionally incorporating conductive fillers like graphene flakes to enhance electrical conductivity.^[^ ^25^, ^26^, ^27^, ^28^ ^]^ Despite these improvements, such viscous LMP inks still require similar post‐treatment procedures, as summarized in **Figure** **1**a. Thus, achieving controllable viscosity suitable for various printing methods without relying on additional processes remains a critical challenge.

In this study, we develop a new LM composite particle (LMCP) ink specifically designed to overcome the long‐standing challenges of conventional LM inks–such as high surface tension, poor wettability, and limited viscosity control–for improved printability and compatibility with scalable 2D coating processes. We then apply this ink formulation to demonstrate its practical utility in stretchable electronics, particularly in the fabrication of metamaterial absorbers (MMAs)–electromagnetic structures composed of periodically patterned electrodes on soft dielectric substrates. MMAs have garnered considerable interest in areas such as cloaking,^[^ ^29^ ^]^ radar,^[^ ^30^ ^]^ sensing,^[^ ^31^ ^]^ light‐emitting diodes,^[^ ^32^ ^]^ and energy harvesting,^[^ ^33^ ^]^ owing to their ability to effectively absorb electromagnetic (EM) waves at target frequencies while being much thinner than conventional absorbers.^[^ ^34^, ^35^ ^]^ Moreover, their EM absorption range can be tailored to multiband, wideband, or ultra‐broadband forms by modifying pattern shapes, material properties, or system architectures.^[^ ^35^ ^]^

Previous efforts to develop stretchable MMAs have primarily relied on either injecting pure LM into microfluidic channels^[^ ^36^, ^37^, ^38^, ^39^, ^40^ ^]^ or using rigid electrode materials such as gold^[^ ^41^ ^]^ and silver.^[^ ^42^ ^]^ While these approaches have demonstrated functional devices, they exhibit inherent limitations: rigid electrodes restrict the strain range and tunability of the resonant frequency, and injection‐based methods often lead to poor resolution, low throughput, and structural unreliability due to issues such as air bubble entrapment in closed‐end channels^[^ ^43^ ^]^ and inconsistent LM volume control. Under these circumstances, printable LM electrodes with high‐resolution and large‐area patterning capabilities offer a compelling alternative. In contrast to prior methods, our printable LMCP ink enables the direct patterning of high‐resolution, large‐area electrode arrays with excellent mechanical stretchability and strain‐dependent electromagnetic tunability, offering a scalable and reconfigurable platform for next‐generation MMA devices.

In this work, we introduce a universally printable LMCP ink that overcomes these challenges by combining low surface tension, high viscosity, and excellent wettability, making it compatible with commercial printing techniques [highlighted in pink (dashed‐line box) in Figure 1a]. This ink facilitates the fabrication of stretchable and flexible LM electrodes with five essential features: coffee‐ring‐free, crack‐free, bilayer‐free, sintering‐free, and uniform dried pattern. These properties are achieved through simple ambient evaporation without the need for additional processing steps.

To meet these requirements, the ink formulation integrates five key elements: i) low surface tension, ii) high wettability, iii) controllable viscosity, iv) self‐sintering capability, and v) uniform final pattern. For (i) and (ii), we use micro‐sized liquid metal particles (LMPs) coated with the dispersant polyvinylpyrrolidone (PVP),^[^ ^44^ ^]^ dispersed in a liquid solvent. To control viscosity (iii), we incorporate Laponite particles as a viscosity enhancer^[^ ^45^ ^]^ and leverage natural sedimentation of the LMPs. For (iv), self‐sintering is facilitated during evaporation by co‐assembling Laponite and PVP on the LMP surface^[^ ^45^, ^46^, ^47^ ^]^ and by adding hydrochloric acid (HCl) to remove the oxide layer on LMPs.^[^ ^19^ ^]^ Finally, to accomplish (v), solutal‐Marangoni mixing flows during binary mixture evaporation contribute uniform distribution of the LMPs across the drying  film.

After optimizing the ink, we evaluate the electrical performance, stretchability, and physicochemical stability of the printed LMCP electrodes and test their compatibility with a range of flexible and stretchable substrates using various commercial printing techniques. We also demonstrate their potential applications in stretchable printed electronics, including sensors, wearable devices, displays, fibers, and energy conversion systems.^[^ ^48^ ^]^ Notably, we focus on their application as stretchable MMAs, which can selectively absorb EM waves in the GHz frequency range, with tunable absorption characteristics achieved through uniaxial stretching.

## 2 Results and Discussion

### 2.1 Design and Performance of Surface Tension‐, Viscosity‐, and Wettability‐Tunable LMCP Inks

Controlling the surface tension (*γ*), viscosity (*µ*), and wettability of the ink is crucial for achieving effective coatability on stretchable and flexible surfaces while ensuring compatibility with commercial printing technologies, as illustrated in Figure 1a. To achieve these properties, we developed the LMCP ink by sonicating bulk LM in a mixture of EtOH and DI water, with a small amount of hydrochloric acid (HCl) added to remove the oxide layer of liquid metal.^[^ ^19^ ^]^ This process converted the bulk LM into micrometer‐sized liquid metal particles (LMPs). The LMPs were then dispersed in a low‐surface‐tension solvent mixture of EtOH and DI water (30:70 vol.%, *γ* ≈ 36.09 mN m^−1^),^[^ ^49^ ^]^ effectively reducing the ink's surface tension and improving its wettability on the substrate. To control viscosity, we added Laponite as a viscosity enhancer and concentrated the LM content by removing the supernatant after natural sedimentation of the LMPs. These steps increased the LM concentration of the ink to approximately 799 mg mL^−1^, which is about 2.5 to 10 times higher than previously reported values.^[^ ^19^, ^46^ ^]^ The resulting viscosity ranged from 10 to 700 cP across shear rates of 10^0^ to 10^3^ s^−1^, significantly surpassing the viscosity of conventional LMP inks [typically ≈O(10^0^ cP)], as shown in Figure S2 (Supporting Information). The optimized ink composition was determined to be LM:Solvent:Laponite:PVP:HCl ≈ 73.671:25.382:0.788:0.158:0.001 wt%. Additionally, the natural sedimentation time (*t*_sed_) was established as approximately 24 h based on empirical optimization studies. All the detailed preparation, fabrication, and measurement procedures are described in the Experimental Section.

Under the specified coating conditions, we successfully fabricated LMCP electrodes by coating or printing the ink onto a stretchable and flexible substrate. The ink was dried at room temperature and atmospheric pressure, as shown in the electrode image in Figure S1(vii) (Supporting Information). These electrodes demonstrate compatibility with various commercial printing methods, as illustrated in Figure 1b. In this study, we primarily utilized nozzle dispensing, brushing, and stencil printing techniques, as they accommodate a wide range of viscosities [≈ O(10^0^‐10^2^ cP)] and low surface tensions [≈ O(10 mN m^−1^)]. The LMCP electrodes produced using this ink exhibited high electrical conductivity [(3.75 ± 2.94) × 10^5^ S m^−1^], comparable to that of pure liquid metal (e.g., Galinstan, 3.46 × 10^6^ S m^−1^),^[^ ^5^ ^]^ despite the inclusion of chemical additives (detailed calculations are provided in the Experimental Section). This remarkable conductivity can be attributed to two key factors: 1) the low content of additives and the high concentration of LMPs, which retain the inherent electrical properties of the liquid metal, and 2) the uniform mixing and effective coalescence of LMPs during evaporation, which form a well‐connected network with short conduction paths. The detailed mechanism underlying this performance will be discussed in the following section.

### 2.2 Evaporation Mechanisms for Uniform Drying and Self‐Sintering Driven by Laponite‐PVP‐LMP Composites

**Figure 2:**
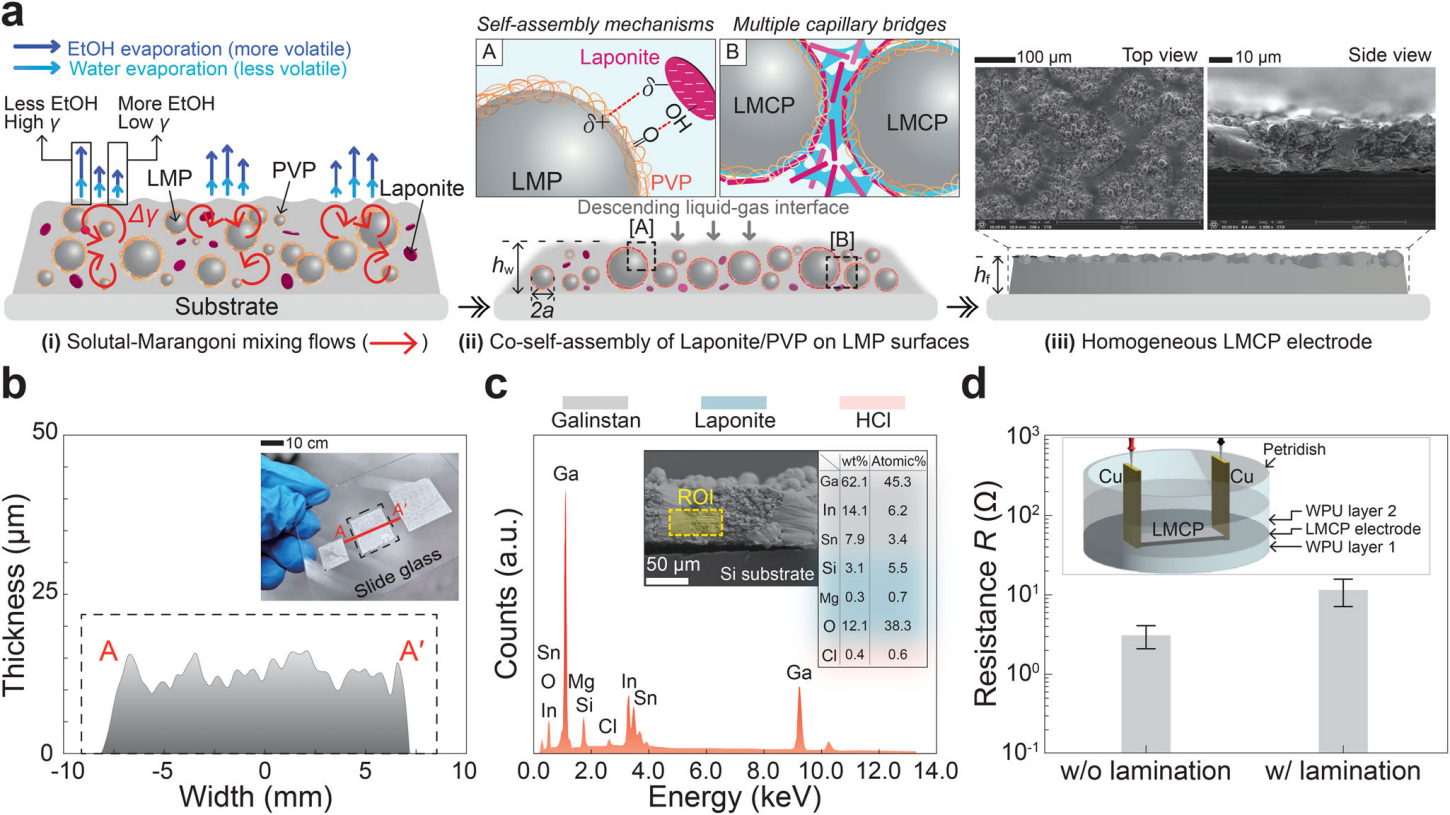
Evaporation‐based coating mechanism of LMCP inks and characterization of the resulting LMCP electrodes. a) Hydrodynamic coating mechanism enabling coffee‐ring‐free, crack‐free, bilayer‐free, and sintering‐free LMCP electrodes. b) Thickness profiles; c) EDS side‐view analysis; and d) Lamination test results. EDS measurement uncertainty is approximately 6.11 ± 1.02%. Detailed information is provided in the Experimental Section.

To understand how LMCP ink achieves high electrical conductivity and uniform drying through simple ambient evaporation, it is essential to examine the evaporation mechanism of the coated/printed ink. This process is summarized in **Figure** **2**a. First [(i) in Figure 2a], during evaporation, the LMPs were uniformly and well dispersed due to the solutal‐Marangoni mixing flows driven by the surface tension gradient along the liquid‐gas interface, induced by the selective evaporation of EtOH.^[^ ^50^, ^51^ ^]^ In this case, the effect of solutal‐Marangoni convective flow predominated over diffusion and viscosity effects during the early stages of evaporation, i.e., Pe (Péclet number) $=LU_{M}/D∼O\left(10^{4}\right)\gg$ 1 and Ca (capillary number) $=\mu U_{M}/\gamma ∼O\left(10^{-3}\right)\ll$ 1, where *L* is the electrode length (≈ 10^−1^ m), *U*_M_ is the typical solutal‐Marangoni flow speed ≈ O(10^−4^ m s^−1^),^[^ ^52^ ^]^ *D* is the diffusion coefficient of the EtOH and DI water mixture solution (≈ 0.5 × 10^−9^ m^2^ s^−1^),^[^ ^53^ ^]^ *µ* is the ink viscosity (≈ 7.0 × 10^2^ cP), and *γ* is the surface tension (≈ 2.5 × 10^−2^ N m^−1^). Here, we assumed that the fluid properties, particularly surface tension and diffusion coefficient, would not be significantly affected by the low concentration of additives (Laponite and PVP, with a combined amount below 1 wt%).^[^ ^54^ ^]^ During ink evaporation, the LMPs well followed the initial flow structures due to St $=2\rho_{p}a^{2}U_{M}/9\mu L\;∼\;O\left(10^{-12}\right)\ll$ 1, where *ρ*_p_ is the LMP density (≈  6440 kg m^−3^), and *a* is the LMP radius (≈ 1µm). In this stage, the sedimentation effect can be neglected, *i*.*e*., *U*_M_/*U*_S_ ≫ 1, where *U*_S_ [= 2(*ρ*_p_ − *ρ*_f_)*a* ^2^ *g*/9*µ*]^[^ ^19^ ^]^ is the settling speed of LMPs (≈ 1.7 × 10^−8^ m s^−1^), where *ρ*_f_ is the fluid density (≈ 935 kg m^−3^), and *g* is the gravitational acceleration (≈ 9.8  m s^−2^).

During the next step [(ii) in Figure 2a], as evaporation progressed, the ethanol (EtOH) component evaporated completely, leaving mostly deionized (DI) water in the ink. This evaporation led to a reduction in the contact angle as the EtOH content decreased, while the wetted contact area on the substrate remained constant. Once the solutal‐Marangoni flows subsided, an outward capillary flow driven by evaporation, commonly known as the coffee‐ring flow,^[^ ^55^, ^56^ ^]^ began to develop. However, due to the high viscosity of the ink, these outward flows were effectively suppressed,^[^ ^57^ ^]^ minimizing the coffee‐ring effect. Furthermore, as the evaporation continued, the ink viscosity increased significantly, causing the sedimentation effect of the particles to diminish, as the sedimentation velocity is inversely proportional to viscosity (*U*_S_ ∝ *µ* ^−1^). In this case, the evaporative flux, driven by the diffusion of the remaining water component into the air, is predominant, *i*.*e*., Pe (= $h_{w}U_{S}/D_{v})∼O\left(10^{-7}\right)\ll 1$. Here, *h*_w_ (≈ *h*_f_/*ϕ*_s_) is the wet film thickness of the printed pattern, where *h*_f_ is the final dry film thickness (≈ 12.8 µm), and *ϕ*_s_ is the LM volume fraction in the evaporating ink (≈ 0.07 after complete evaporation of EtOH). *D*_v_ is the diffusion coefficient of water vapor into air (≈ 2.2 × 10^−5^ m^2^ s^−1^).^[^ ^58^ ^]^ Therefore, instead of sedimentation dominating, the LMPs settled uniformly onto the substrate, following the gradual descent of the liquid‐gas interface. This process ensured uniform drying of the electrode and effectively prevented the formation of coffee rings.

As the concentration of Laponite nanoplates reached a critical threshold, they began to self‐assemble on the surfaces of LMPs.^[^ ^45^, ^46^ ^]^ During this process, the PVP molecules that initially capped the LMP surfaces bound to the Laponite structure,^[^ ^47^ ^]^ resulting in the formation of liquid metal composite particles (LMCPs) encapsulated by Laponite and PVP. These self‐assemblies were primarily driven by two interfacial interactions: 1) electrostatic attraction between the positively charged (δ^+^) LMPs with their PVP capping layer (as verified by *ζ*‐potential analysis in Figure S3a, Supporting Information)^[^ ^19^ ^]^ and the negatively charged (δ^−^) surfaces of Laponite, and 2) hydrogen bonding between the carbonyl groups of PVP and the hydroxyl groups on the Laponite surface.^[^ ^59^, ^60^, ^61^ ^]^ The Laponite layer on the LMCP surface exhibited highly hydrophilic properties (see Figure S4, Supporting Information), facilitating the formation of capillary bridges. As a result, LMCPs could form multiple capillary bridges, as illustrated in inset B of Figure 2a(ii), allowing suspended particles to adhere to each other and merge.^[^ ^62^ ^]^ Assuming a pair of 1 µm‐sized LMCPs are 10 nm apart, the capillary force between them is approximately 0.72 nN (see Figure S5, Supporting Information). As the ink evaporates, the particles move closer, causing the capillary force to increase substantially, eventually reaching ≈ O(10 nN). Upon contact, the contact area approaches zero, generating extremely high compressive pressure, on the order of GPa levels.^[^ ^63^ ^]^ This intense pressure fuses the LMCPs into a single structure, as depicted in Figure 2a(iii).

Through this evaporation process, we fabricated LM electrodes that were coffee‐ring‐free, crack‐free, bilayer‐free, and sintering‐free, as shown in Figure 2b–d. The LMCPs dried and agglomerated uniformly, resulting in a film thickness of 12.8 ± 3.7 µm (see Figure 2b). Side‐view EDS analysis confirmed that the Laponite additive was evenly distributed with the LM components, attributed to the high LM‐to‐liquid ratio of the ink achieved by removing the supernatant during the natural sedimentation process (see Figure 2c).^[^ ^46^ ^]^ Furthermore, even after lamination with water‐borne polyurethane (WPU), the electrode maintained its crack‐free surface and stable electrical conductivity (see Figure 2d). A slight increase in resistance was observed, likely caused by infiltration of the WPU solution between the LMCP electrode and the copper tape, increasing the contact resistance.

It is important to highlight that this uniform and conductive network is achieved entirely under ambient conditions without requiring any thermal or mechanical post‐treatment. While previous studies have used PVP primarily to provide steric stabilization of LM droplets or nanoparticles,^[^ ^14^, ^64^, ^65^ ^]^ our formulation uniquely exploits the cooperative self‐assembly of PVP and Laponite on LM surfaces during solvent evaporation. This hybrid shell facilitates capillary bridge formation between LM particles, thereby promoting spontaneous self‐sintering and robust electrical percolation. Such a mechanism distinguishes our approach from prior gel‐based or ligand‐modified inks, offering a scalable pathway toward printable, durable stretchable electrodes.

### 2.3 Versatile Printing Application with Ultra‐Stretchability, Long‐Lasting Durability, and Superior Wettability

**Figure 3:**
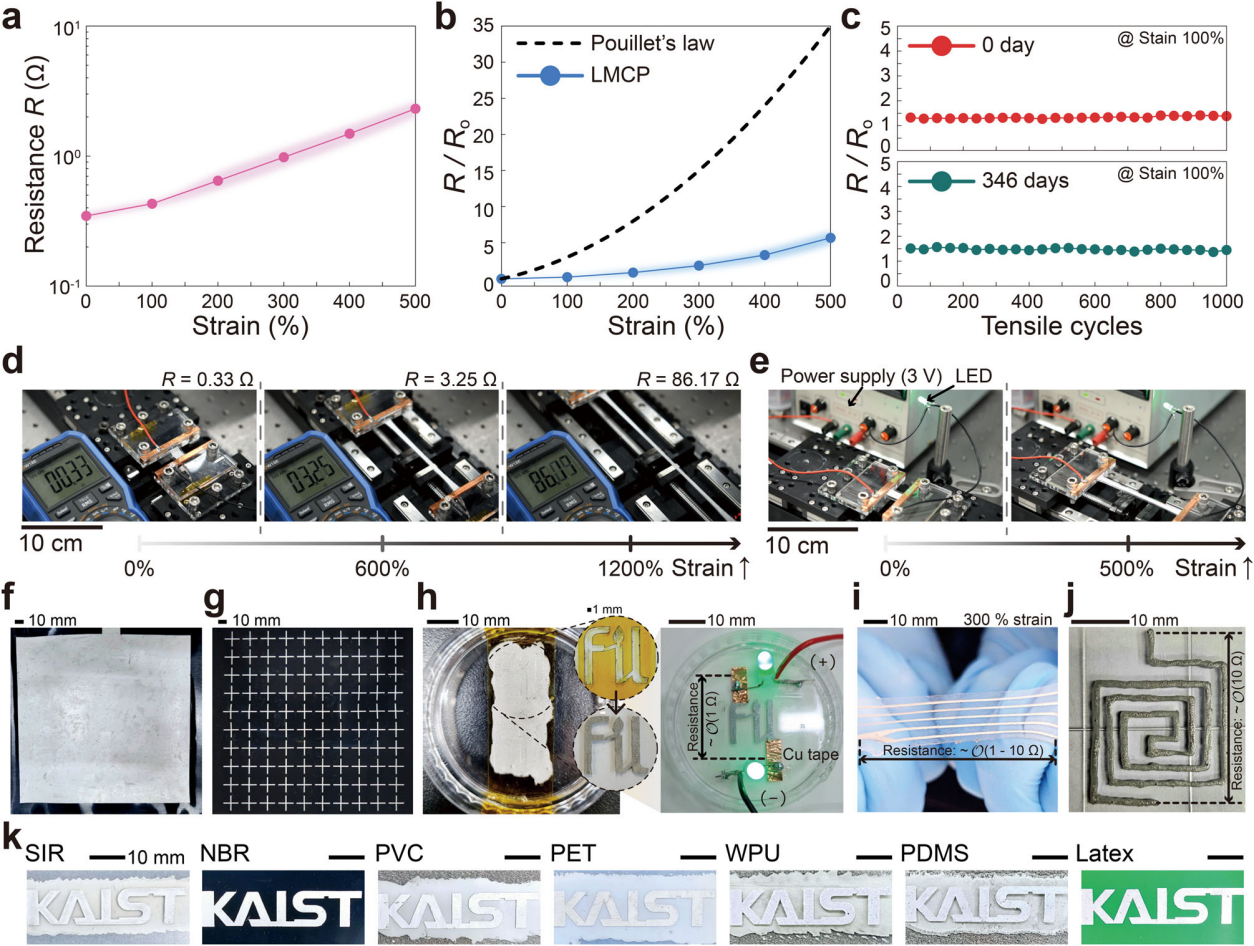
Ultra‐stretchability and extensive design tunability of LMCP patterns on various substrates. a) Electrical resistance change and b) relative resistance variation under uniaxial deformation of the substrate, up to 500% strain. c) Cyclic stretching and relaxation tests (1000 cycles at 100% strain) on electrodes at 0 and 346 days after fabrication, both without a lamination layer. *R*_o_ and *R* represent the resistance at zero strain and the resistance that varies with strain, respectively. Here, the shaded areas indicate the error margins. d) Conductivity test of LM electrodes under ultra‐elongation conditions (0‐1200% strain). e) Stretchable LED operating under 0–500% strain. f) Large‐area square pattern (20 cm × 20 cm) and g) cross‐shaped multi‐array (10 × 10) patterns on Nitrile‐butadiene rubber (NBR) substrates. The square‐ and cross‐shaped array patterns were printed using brushing and stencil techniques, respectively. h) Stencil‐printed laboratory symbol with a polyimide shadow mask. The LED on/off test was performed with a voltage of 3.5 V. i) Stretchable zigzag U‐shaped electrode circuit. j) LMCP circuit patterns fabricated with a 240 µm diameter needle. k) University symbol patterns printed on diverse stretchable and flexible substrates, each showing resistance on the order of 1–10 Ω. Here, all symbol patterns were printed using the stencil method.

Building on the optimized evaporation mechanism and ink composition, we evaluated the performance and versatility of LMCP electrodes under various conditions. Specifically, we tested the electrical resistance, stretchability, and durability of square‐shaped LMCP electrodes (3 cm × 1 cm) printed on WPU substrates under the same evaporation conditions (**Figure** **3**a–e). Without any applied strain, the electrodes exhibited a low resistance of approximately 0.35 Ω. When stretched uniaxially from 0% to 500% strain, the resistance increased monotonically, as shown in Figure 3a (see also the Δ*R*/*R* data in Figure S6, Supporting Information).

According to Pouillet's law, *R* = *ρ*(*L*/*A*) = *R*_o_(1 + *ɛ*)^2^, assuming an incompressible conductor (where *ρ* is the resistivity, *L* is the electrode length, *A* is the cross‐sectional area, *R*_o_ is the initial resistance, and *ɛ* is the strain, Δ*L*/*L*),^[^ ^66^ ^]^ the resistance should sharply increase due to the elongation of *L* and the corresponding reduction in *A* (see dashed line in Figure 3b). However, even under 500% tensile strain, the electrodes maintained resistance within a few ohms (see the pink line in Figure 3a) and exhibited a relative resistance ratio below ten (see the bluish line in Figure 3b). This exceptional performance is attributed to the fully merged LMCP structure, as observed in the SEM images of Figure 2a(iii), which created multiple interconnections of conductive paths capable of accommodating significant deformation. These interconnections are not merely the result of physical contact, but are formed through metallic reconstruction during the capillary‐force‐driven self‐sintering process, ensuring stable electrical conduction under mechanical deformation (see Figure S7, Supporting Information for sintering dynamics).

This percolated structure, combined with the intrinsic fluidity of LM, likely suppresses cross‐sectional thinning during stretching. As a result, the resistance remains significantly lower than the theoretical value predicted based on purely geometric deformation under constant volume assumptions. Specifically, at 500% strain, the experimental *R*/*R*_o_ was 6.82 ± 0.79, in contrast to the theoretical value of 36 (see Figure 3b), suggesting that the intrinsic fluidity of LM and the robust interconnections formed during sintering help preserve effective conduction pathways even under extreme deformation.

During cyclic stretch‐and‐release testing (Figure 3c), the LMCP electrodes consistently maintained Δ*R*/*R* values below 1.5 over 1000 cycles at 100% strain immediately after fabrication (see red circle symbols in Figure 3c). Remarkably, even after 346 days of ambient air exposure, the electrodes demonstrated excellent physicochemical stability and electrical performance without requiring additional protective layers (see green circle symbols in Figure 3c). This long‐term stability is likely due to the solid‐like LMCP structure, which minimizes exposed surface area and limits air‐induced reactions, as illustrated in Figure 2a(iii).

Additionally, the electrodes maintained conductivity under extreme stretching conditions, enduring up to 1200% elongation (Movie S1, Supporting Information). While resistance increased under such extreme deformation (see Figure 3d; Figure S8, Supporting Information), the electrodes also demonstrated self‐healing capabilities after breaking, as shown in Figure S9 (Supporting Information). To further assess the feasibility of the electrodes, we conducted a light‐emitting diode (LED) performance test (Figure 3e; Movie S2, Supporting Information).

We also fabricated large‐area patterns spanning tens of centimeters and fine patterns ranging from several hundred micrometers to a few centimeters, including complex designs such as crosses, symbols, and circuits (Figure 3f–j; Movie S3, Supporting Information). These patterns were created using three printing techniques–brushing, stencil printing, and nozzle dispensing–each requiring specific ink viscosities and surface tensions (*e*.*g*., *µ* ⩾ 100 cP for brushing and stencil printing, and *µ* ⩾ 5 cP with 28 ⩽ *γ* ⩽ 40 mN m^−1^ for nozzle dispensing).^[^ ^7^, ^11^, ^67^ ^]^ In particular, using stencil printing, we successfully achieved fine patterning down to ≈ O(10 µm), demonstrating the ink's suitability for high‐resolution applications (see Figure S10, Supporting Information).

Furthermore, LMCP inks were successfully patterned on various stretchable and flexible substrates, as shown in Figure 3k. The resulting patterns exhibited strong adhesion to diverse substrates, with adhesive strength measured at approximately 28.7 kPa in lap shear tests on glass over 24 h (see Figure S11, Supporting Information). This strong adhesion is attributed to hydrogen bonding between hydroxyl groups on the substrate surface and the carbonyl groups of PVP and hydroxyl groups in Laponite. Consequently, LMCP patterns are expected to exhibit strong adhesion to substrates with hydroxyl groups, either naturally present or introduced via surface treatments such as plasma treatment or chemical functionalization.

### 2.4 Potential Applications: Stretchable Metamaterial Absorbers

Liquid metal (LM) has recently gained attention as a flexible electrode material for developing deformable metamaterial absorbers (MMAs). However, most existing LM‐based MMAs rely on intricate microfluidic channels fabricated by molding or 3D printing, which limit scalability and suffer from reliability issues such as air entrapment and structural deformation.^[^ ^36^, ^37^, ^38^, ^39^, ^40^ ^]^ In this study, we overcome these limitations by introducing, to the best of our knowledge, the first fully printed and stretchable MMA system based on LMCP electrodes. Utilizing ambient, planar printing techniques, our approach enables the fabrication of large‐area, complex‐shaped MMA patterns (Figure 3f–i), offering significant advantages in manufacturability, scalability, and application diversity for next‐generation electromagnetic (EM) devices.

**Figure 4:**
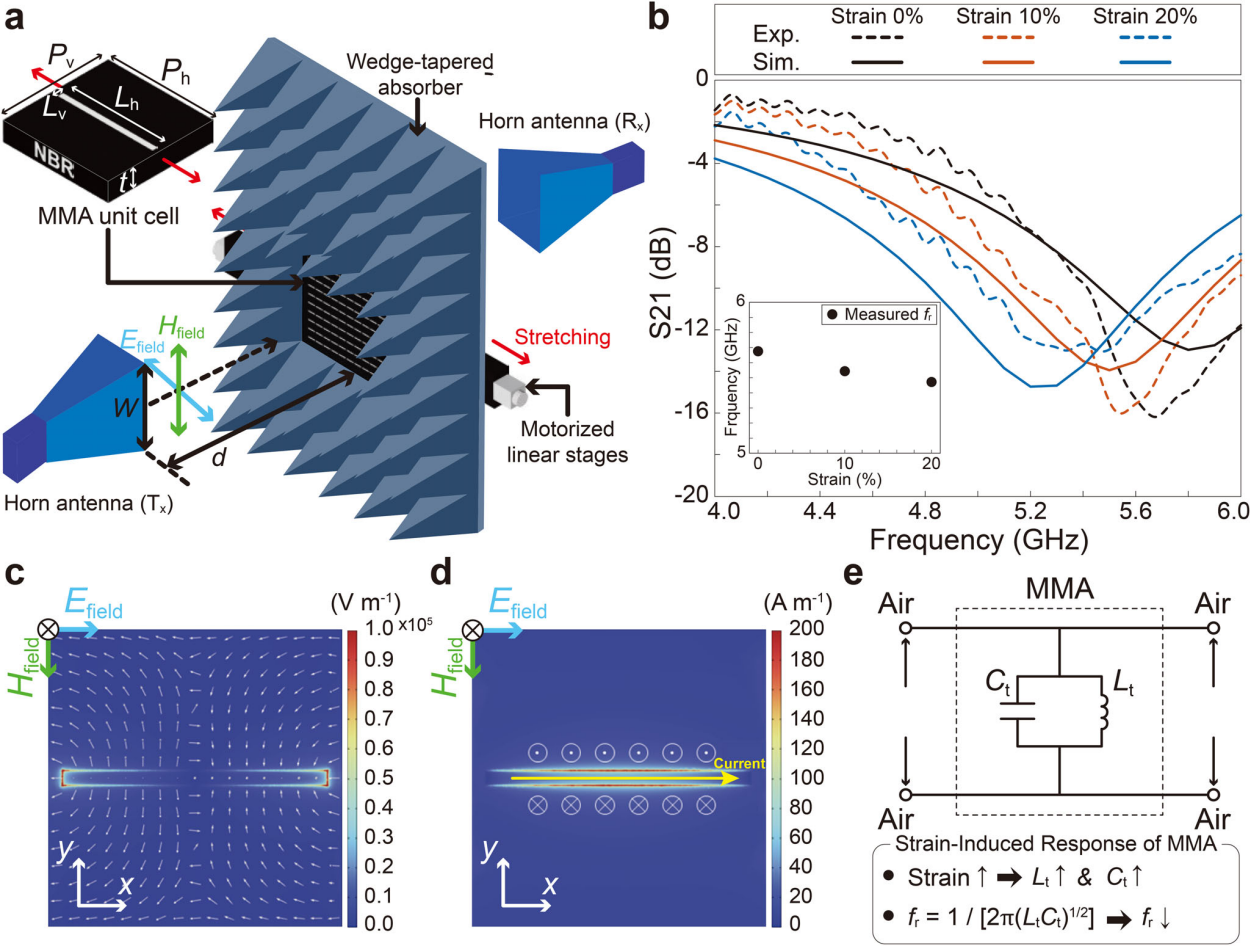
Stretchable metamaterial absorber (MMA) consisting of multi‐array LMCP patterns. a) Schematics of the experimental apparatus for testing a resonant absorptivity of fabricated MMA. Illustration of the unit cell is provided, and detailed parameters in (a) are described in the main text. Transmitter (T_x_) and receiver (R_x_) antennas faced each other in a straight line, and MMA attached to a motorized linear stage was placed vertically between them. b) Comparison between measured and simulated transmission curves in the frequency range of 4–6 GHz, depending on the tensile strain up to 20%. Transmission coefficient S21 expressed as S21 = 10log_10_(*P*_trans._/*P*_inc._), where *P*_trans._/*P*_inc._ represents the ratio of the transmitted power to the incident power, was obtained from the vector network analyzer. c) Simulated electric field intensity/vector distribution and d) magnetic field strength distribution in the unit cell induced by the given *E*_field_ and *H*_field_ of wave. The white vectors in (c) represent the magnitude and orientation of the electric field. The yellow arrow and cross/dot symbols in (d) indicate the typical current flow direction and the magnetic field orientation, respectively. Only the results for a single electrode are presented here. e) Equivalent circuit for (c) and (d). Here, *C*_t_ and *L*_t_ mean the total capacitance and inductance of the MMA system, respectively. Detailed information about the simulation and experimental procedures is provided in the Experimental Section.

We then experimentally and numerically investigated the shift in resonant frequency (*f*_r_) of the LMCP‐based MMAs under uniaxial stretching (**Figure** **4**a,b). Experimentally, in the unstretched state, the MMA exhibited a resonant frequency of approximately 5.68 GHz, selectively absorbing electromagnetic (EM) waves at this frequency. When stretched along the longer axis of the electrodes, the resonant frequency progressively decreased: from 5.68 GHz at 0% strain to 5.54 GHz at 10% strain, and further to 5.47 GHz at 20% strain (see dashed lines and inset of Figure 4b). These experimental results nicely showed a good agreement with the wave simulation data in terms of the strain‐dependent frequency shift, confirming that the resonant frequency decreases consistently with increasing strain. Note that discrepancies between experimental and simulation data are likely due to differences in array size, strain, and the dielectric properties of the NBR substrate. For instance, while the simulation assumes an infinite array of perfectly periodic MMA unit cells under homogeneous strain in the MMA substrate, the experimental setup involves a finite 10 × 10 array, where imperfections in the sample holder used in the experiments may cause non‐negligible slippage, leading to non‐uniform substrate stretching. Despite these differences, the results demonstrated that the absorption band of EM waves in the GHz frequency range can be systematically tuned by applying uniaxial strain to the LMCP‐based MMA, while maintaining high absorptivity. Additionally, the measured spectra at different strains retained consistent quality factors (*Q*‐factors, *Q* = *f*_r_/FWHM), indicating that the LMCP electrodes preserve their performance under deformation. Here, the *Q*‐factor is defined as the ratio of the resonant frequency to the full width at half maximum (FWHM).

To better understand the strain‐induced shift in resonant frequency, we analyzed the electric field intensity/vector distribution and magnetic field strength using 3D electromagnetic (EM) simulations (Figure 4c,d). When the incident EM wave's electric field oscillation (*E*_field_) was aligned horizontally (*x*‐direction) with the electrodes, as indicated by cyan arrows in Figure 4c,d, free electrons moved along the electrode surfaces in the *x*‐direction. This movement induced a strong localized electric field on the MMA,^[^ ^68^, ^69^ ^]^ as shown by the arrows in Figure 4c. The electron flow also generated a current in the *x*‐direction, which in turn induced a magnetic field, as illustrated in Figure 4d.

The MMA's behavior can be modeled as an equivalent circuit (Figure S12, Supporting Information). Each electrode exhibits self‐capacitance (*C*_s_), while interactions with neighboring unit cells in the *x*‐direction contribute mutual capacitances (*C*_m_). Since the electric field induced on one electrode aligns with that of adjacent electrodes, the total capacitance is expressed as *C*_t_ = *C*_s_ + *C*_m_/2. Similarly, each unit electrode has self‐inductance (*L*_s_), with mutual inductance (*L*_m_) arising from interactions with adjacent electrodes in the *y*‐direction. Because the magnetic fields generated by neighboring electrodes oppose each other, the total inductance is expressed as *L*_t_ = *L*_s_ − 2*L*_m_.^[^ ^70^ ^]^

The resonant frequency (*f*_r_) of the MMA is determined by the equivalent circuit parameters, as described by the electrical circuit model in Figure 4e, and is expressed as $f_{r}=1/2\pi \sqrt{L_{t}C_{t}}$. At resonance, the MMA's surface impedance approaches that of free space, enabling maximum EM absorption. When the MMA is stretched horizontally, both the total inductance (*L*_t_) and capacitance (*C*_t_) increase due to the elongation of the electrodes.^[^ ^70^ ^]^ Consequently, the resonant frequency decreases according to *f*_r_ ∝ (*L*_t_ *C*_t_)^−1/2^. This behavior is consistent with the experimental results in Figure 4b, where the resonant frequency decreases systematically with increasing strain.

## 3 Conclusion

In this study, we developed a liquid metal composite particle (LMCP) ink with tunable surface tension, high viscosity, and excellent wettability, specifically designed for compatibility with various stretchable and flexible substrates and integration into commercial printing technologies. The ink composition consisted of liquid metal (LM) (73.671%), solvent (25.382%), Laponite (0.788%), PVP (0.158%), and HCl (0.001%) by weight, with a natural sedimentation time of approximately 24 h. Practical experiments demonstrated the ink's capability to create patterns at multiple scales–from large designs spanning tens of centimeters to fine features as small as hundreds of microns–using standard commercial printing methods such as nozzle dispensing, brushing, and stencil printing. Upon drying, the LMCP ink exhibited uniform drying properties, free from coffee‐ring effects, cracks, or bilayer formations, and formed electrically conductive electrodes via a self‐sintering process under ambient conditions, eliminating the need for complex manufacturing steps or specialized equipment.

The resulting LMCP electrodes showcased three outstanding features: i) high electrical conductivity of approximately (3.75 ± 2.94) × 10^5^ S m^−1^, ii) exceptional stretchability beyond 1200% strain, and iii) long‐term physicochemical durability, retaining performance for over 346 days even under continuous exposure to ambient air without protective coatings. Furthermore, we demonstrated the potential of LMCP electrodes as stretchable metamaterial absorbers (MMAs), a promising application in next‐generation stretchable and flexible electronics. When printed in a multi‐array format on a stretchable dielectric substrate, the LMCP‐based MMAs selectively absorbed electromagnetic (EM) waves in the 5–6 GHz range, with precise tuning achieved through uniaxial stretching at strains between 0% and 20%. This tunability, combined with the ease of large‐scale printing and superior stretchability, offers significant advantages over conventional stretchable MMA technologies.^[^ ^36^, ^37^, ^38^, ^39^, ^40^, ^41^, ^42^ ^]^ Additionally, the viscosity of the LMCP ink can be tailored by adjusting the Laponite concentration and sedimentation time to meet the specific requirements of different printing methods, enhancing its versatility. This adaptability positions the LMCP ink as a highly promising material for diverse applications in stretchable and flexible electronics, including on‐body devices, soft robotics, energy conversion and storage systems, and nanogenerators.^[^ ^71^, ^72^ ^]^

## 4 Experimental Section

### Material

For this study, Galinstan fluid 4N, purchased from Geratherm Medical AG, Germany, composed of 68.5 wt% gallium, 21.5 wt% indium, and 10.0 wt% tin was used. Ethanol (200 proof, anhydrous ≫ 99.5%), hydrochloric acid [37% (w/w) ≈ 12 M], and polyvinylpyrrolidone (average molecular weight: 40 000 g mol^−1^) were all purchased from Sigma–Aldrich. Laponite (Laponite RD, BYK‐Chemie GmbH, Germany) was utilized. Laponite RD, a hydrous sodium lithium magnesium silicate with the chemical formula [Mg_5.5_Li_0.3_Si_8_O_20_(OH)_4_Na_0.7_], features a disc‐shaped structure with a diameter of ≈ 25 nm and a thickness of ≈ 1 nm. When dispersed in a liquid, Laponite particles were negatively and positively charged at their surface and edge, respectively.^[^ ^73^ ^]^ Information of the substrate used in all the experiments was as follows: Silicone rubber, SIR (Apple Silicone, South Korea); Nitrile‐butadiene rubber, NBR (GFgjd0002, Gefang Silicone Products, China); Polyvinyl chloride, PVC (Filmbank, South Korea); Latex (Spoholic, South Korea); Polyethylene terephthalate, PET (NB‐TP‐3GU100, Mitsubishi Paper Mills Limited, Japan); Water‐borne polyurethane, WPU (Akuarane 3410/26302, T&L, South Korea).

### Sample Preparation

The fabrication steps of the LMCP ink were described in the following (see Figure S1, Supporting Information): i) Initially, 500 µL of bulk liquid metal was added to a 10 mL mixture of ethanol (EtOH) and deionized water (30:70 vol.%). The EtOH concentration was determined to be the maximum level at which Laponite can be stably dispersed.^[^ ^54^ ^]^ ii) Next, an ultrasonic homogenizer (KUS‐650, KBT, South Korea) operating at 350 watts was used to create the LMPs in the mixture solution. The homogenizer was operated intermittently for 3 min with a cycle of 2 s on and 4 s off. During the sonication, the solution was cooled in an aluminum chamber placed on a dry bath chiller (H2O3‐96, Ginkgo Biotechnology, USA). iii) After the sonication, 20 mg of polyvinylpyrrolidone (PVP) was added to stabilize the dispersity of LMPs, 100 mg of Laponite nanoplate powder as a viscosity‐enhancing agent, and 50 µL of hydrochloric acid (HCl) to remove the oxide layer on the LMP, which induced its self‐sintering properties, respectively, into the 10 mL mixture solution. The HCl volume (50 µL) was selected based on theoretical estimation as the minimum amount required to completely remove the Ga_2_O_3_ oxide shell from the surface of the dispersed LM particles in the 10 mL ethanol–water mixture (see Note S1, Supporting Information). iv) Subsequently, the sonication was performed again with the same operational conditions to ensure uniform mixing. During this mixing process, HCl effectively removed the oxide layer from the surface of the LMPs, while facilitating the efficient self‐assembly of PVP onto the LMPs (evidenced by the increase in particle size and enhancement in *ζ*‐potential, as shown in Figure S13, Supporting Information).^[^ ^74^ ^]^ v) To further increase the viscosity of the ink, the liquid solvent and LM particles were separated by natural sedimentation based on density difference, allowing the mixture to settle for about 24 h. vi) As a final step, the supernatant was selectively extracted by using a syringe, and the remaining LMCP ink was mixed well using a vortex mixer for 30 s.

To fabricate a liquid metal pattern, a patterned mask was prepared using polyimide tape affixed to a substrate. The tape was precisely cut into the desired shapes with a VLS 3.5 laser cutter, with the laser parameters set to 35% power for 0.05 mm‐thick copier paper to minimize substrate damage. Before applying the LMCP ink, the substrate underwent surface treatment using a BD‐10ASV corona treater (Electro‐Technnic Products, USA) to improve ink wettability. For polydimethylsiloxane (PDMS) substrates, the base elastomer and curing agent were mixed in a 10:1 weight ratio (Sigma–Aldrich, USA). Air bubbles were removed from the mixture using a vacuum chamber, and the PDMS was cured at 22°C for over 24 h to ensure complete degassing. This was followed by baking at 60°C in a forced convection oven (OF‐22GW, JEIOTECH, South Korea) to complete the curing process. The same procedure was applied to fabricate WPU substrates.

### Optimization for LMCP Ink Formulation

The LMCP ink composition was optimized by adjusting the concentration of Laponite and PVP, as shown in Figure S14 (Supporting Information). As the Laponite concentration was increased, its solvent‐trapping effect became more pronounced, effectively preventing the natural sedimentation of LMPs (see the first row in Figure S14, Supporting Information). This hindered the subsequent removal of the supernatant, which was a crucial step in increasing the viscosity of the LMCP ink [see Figure S1(vi), Supporting Information]. Conversely, higher PVP concentrations improved the dispersibility of LMPs by increasing the steric repulsion between particles (see the first column in Figure S14, Supporting Information). To achieve good dispersion and efficient sedimentation, the optimal concentrations of Laponite (100 mg) and PVP (20 mg) were empirically found. These concentrations minimized the reduction in the inherent electrical conductivity of the LM while still allowing for sufficient natural sedimentation of LMPs.

During the natural sedimentation process [(v) step in Figure S1, Supporting Information], Laponite can trap the liquid solvent through its self‐assembled structure.^[^ ^75^ ^]^ It had been reported that Laponite nanoparticles could form a ‘house‐of‐cards’ (HOC) structure due to their plate‐like shapes and electrostatic interactions between the particles when they were suspended in the liquid solution, as depicted in Figure S14 (Supporting Information).^[^ ^54^, ^75^, ^76^ ^]^ When the ink was prepared through natural sedimentation without Laponite (see the reddish box in Figure S2, Supporting Information), the solvent and LMP layers were almost completely separated. In this situation, the insufficient amount of solvent in the final ink prevented the effective capillary bridging of the particles, leading to incomplete merging during evaporation. As a result, the tensile forces induced by constraints imposed by the underlying substrate during drying, which opposed the inward capillary forces, could not be sufficiently relaxed, resulting in severe cracks,^[^ ^77^, ^78^ ^]^ as shown in Figure S15 (Supporting Information). Furthermore, the rapid evaporation caused by the low liquid concentration accelerated the formation of cracks.^[^ ^79^ ^]^

In the experiment, it was observed that as the sedimentation time increased, the solvent‐trapping effect of the Laponite particles gradually weakened, as seen in Figure S16 (Supporting Information). This likely occurred because gravity caused the Laponite's HOC structures to settle with the LMPs, leading to a denser HOC structure and an increase in the local Laponite concentration.^[^ ^80^, ^81^ ^]^ As the Laponite particles moved closer together, as in this situation, the electrostatic repulsion forces between face‐to‐face or edge‐to‐edge (H‐bond) interactions became significant. On the other hand, the edge‐to‐face (T‐bond) interactions, which were critical for maintaining the HOC structures, weakened under these conditions. This ultimately destabilized the HOC network^[^ ^82^ ^]^ and reduced its capacity to trap solvent. From this result, it was noticed that the solvent‐trapping effect of Laponite is time‐dependent. To determine the optimal natural sedimentation time (*t*_sed_), Energy‐Dispersive X‐ray Spectroscopy (EDS) analysis was conducted on LMCP electrodes prepared with sedimentation times of 3, 24, and 288 h (Figure S17, Supporting Information). The EDS results showed that the concentration of LM components (Ga, In, Sn) increased with longer sedimentation times, while the amounts of HCl (Cl) and Laponite (Si, Mg, O) in the LMCP ink decreased significantly. This reduction occurred because, as sedimentation time increased, more solvents–including HCl and Laponite–were removed during the ink production process [(vi) step in Figure S1, Supporting Information]. The Cl component was formed via a chemical reaction between HCl and the oxide layer (Ga_2_O3) on the surface of LMPs, following the reaction: Ga_2_O_3_ + 6HCl → 2GaCl_3_ + 3H_2_O. Insufficient HCl and Laponite in the finalized ink prevented the effective removal of the oxide layer and hindered the proper formation of LMCPs during ink evaporation, leading to crack formation. At the longest sedimentation time (*t*_sed_ ≈ 288 h), partial cracks were observed, and minimal HCl and Laponite were detected near the crack areas. Dynamic Light Scattering (DLS) results (Figure S3b, Supporting Information) further supported this finding. As sedimentation time increased, the particle size distribution shifted toward smaller sizes, likely due to a weakening of the merging force caused by the reduced effectiveness of HCl in removing the oxide layer. Based on these analyses, 24 h as the optimal sedimentation time was identified.

### Characterization of LMCP Ink and Electrode Properties

To prove its versatility for diverse printing methods, it was important to define the properties of the LMCP ink. To measure its viscosity, a rheometer (MCR 501, Anton Paar, Austria) with a cone‐and‐plate geometry (CP50‐1, Anton Paar, Austria) was utilized. The initial ink volume for all cases was 10 mL. The shear rate was set to range from 10^0^ to 10^3^ s^−1^. Here, comparisons were conducted among three cases: i) conventional LMP ink, ii) conventional LMP slurry ink, and iii) LMCP ink. The viscosity measurement results displayed a range of roughly 4 to 20 cP, 40 to 1200 cP, and 10 to 700 cP, respectively (see Figure S2, Supporting Information). By using a centrifuge (1236R, LaboGene, Denmark) with 282 G for 10 min, the LM concentration in the LMCP ink was measured. The measurement result showed that the LM concentration in the LMCP ink was estimated to be 799 mg mL^−1^.

The particle size distribution and *ζ*‐potential of the ink was measured, as shown in Figures S3 and S13 (Supporting Information), using a nanoparticle size analyzer (Zetasizer Nano‐ZS, Malvern Panalytical, UK). Measurements were conducted under two conditions: 1) with and without the second sonication step described in Figure S1(iv) (Supporting Information) and 2) at different sedimentation times (*t*_sed_ ≈ 3, 24, and 288 h). The ink was diluted 100‐fold and placed in a disposable collapsible capillary cell (DTS1070, Malvern Panalytical, UK). Based on the measurements at different sedimentation times, the LMP diameter and *ζ*‐potential value are 3.2 ± 0.7 µm and 19.2 ± 0.9 mV for *t*_sed_ ≈ 3 h, 2.2 ± 0.5 µm and 22.2 ± 0.6 mV for *t*_sed_ ≈ 24 h, and 1.3 ± 0.2 µm and 29.0 ± 0.9 mV for *t*_sed_ ≈ 288 h, respectively. The slight increase in *ζ*‐potential with decreasing LMP size (as sedimentation time increased) likely resulted from enhanced interparticle interactions due to a higher surface‐to‐volume ratio. Under optimal conditions (*t*_sed_ ≈ 24 h), the LMCP ink maintained stable dispersion with a positive *ζ*‐potential of approximately 22.2 ± 0.6 mV. The pH of the LMCP ink was also measured to be 2.61 ± 0.04 using a portable pH meter (Orion Star^*TM*^ A221, Thermo Fisher Scientific Inc., USA), calibrated with standard buffer solutions at pH 4.00, 7.00, and 10.00. The calibration yielded an average slope accuracy of 96.7%.

For investigating the physicochemical properties of the LMCP electrode, an environmental scanning electron microscope (Quattro S, FEI, USA) was utilized to observe high‐resolution images [see Figure 2a(iii)] and conduct EDS measurements (see Figure 2c). During the EDS evaluation, X‐ray energies below 10 keV were used. Additionally, to evaluate the electrical performance of the LMCP electrodes, the electrical conductivity (*σ*) was estimated based on the sheet resistance (*R*_s_) and the final dry thickness of the electrode (*h*_f_). An electrode sample was prepared on a glass substrate for measurement (see the inset of Figure 2b). The sheet resistance was measured using an in‐line four‐point probe (SEMI MF1529) connected to a parameter analyzer (4200‐SCS, Keithley, USA) with a probe (M4P205, MSTECH, South Korea). The electrode thickness, approximately 10 µm, was determined using a confocal laser scanning microscope (VK‐X1050, Keyence, Japan; see Figure 2b). Using the formula *σ* = (*R*_s_ *h*_f_)^−1^, the electrical conductivity was calculated to be around (3.75 ± 2.94) × 10^5^ S m^−1^.

For the stretching and cyclic stretch‐and‐release tests, a digital multimeter (OW18E, OWON Technology, China) was utilized to measure the real‐time resistance of the LM electrodes. To prevent physical damage caused by direct contact with the multimeter probes, 3M 1181 copper tape (3M, USA) was used to make a 1 cm contact with both ends of the LM electrode. The copper tape was wrapped around an acrylic plate and firmly secured with screws, ensuring tight contact and minimizing contact resistance between the electrode and the copper tape, as shown in images of Figure 3d–e. All electrical conductivity tests were conducted at an ambient temperature of 23.0 ± 0.5°C and a relative humidity below 60%.

### Experiments and Simulations of Stretchable Metamaterial Absorbers

Based on the characterization and fabrication of the LMCP electrodes, their practical application to a stretchable MMA was demonstrated. The LMCP‐based stretchable MMA consisted of a hundred rectangular patterns with an arrangement of 10 × 10, while a single pattern had dimensions of 1 mm × 8 mm (vertical length *L*_v_ × horizontal length *L*_h_), as depicted in Figure 4a. A digital photograph of the printed MMA pattern is also provided in Figure S18 (Supporting Information). They were printed on a 1‐mm‐thick dielectric substrate of NBR with vertical and horizontal periods of *P*_v_ = *P*_h_ = 20 mm. Note that the printed pattern formation with the dielectric properties determines the MMA's characteristics (here, a resonant frequency), and the test sample was designed to set the resonant frequency within the target frequency range of 4–6 GHz. Tunable characteristics of the stretchable MMA regarding the microwave were investigated using the experimental setup shown in Figure 4a and validated by the numerical simulation, as displayed in Figure 4b. Two identical horn antennas (HS‐18720A‐NF, Fei Teng Wireless Technology, Taiwan), of which operating frequency ranges from 3.90 to 5.85 GHz with a gain of 20 dBi, were used as a transmitting and receiving antenna array. The electric field of the incident EM wave was aligned parallel to the horizontal direction of the patterns in the MMA. The distance *d* between the stretchable MMA and the antennas was set to satisfy the far‐field condition, which is determined by *d* = 2*W* ^2^/*λ*, to prevent any wave distortion (see Figure 4a). Here, *W* is one of the edges of the antenna exit, and *λ* is the wavelength of the EM wave. In this study, given that the edge of the antenna exit was 212 mm and the target frequency was near 5 GHz, the distance between the stretchable MMA and the antenna was set to more than 1.50 m. A wedge‐tapered absorber (KSS‐ABS4, Korea shield system, South Korea) was placed around the MMA to prevent reflected waves from the surrounding environment, as illustrated in Figure 4a. The transmission coefficient of the MMA was acquired using a vector network analyzer (eVNA‐63+, Mini‐Circuits, Israel) equipped with the antennas at 4–6 GHz by sampling 1000 points. An FFT filtering with a cutoff sampling frequency of 1.11 × 10^−8^ s and a 15‐point window was applied to the measured spectrum.

3D EM wave simulation was performed to validate the experimental results of the stretchable MMA in COMSOL Multiphysics 6.1.^[^ ^83^ ^]^ The finite element method (FEM) was used to solve the governing equations of the EM wave module in COMSOL, and the electromagnetic response of the stretchable MMA was simulated in the frequency domain. To simplify the analysis, the 10 × 10 electrode patterns were approximated as an infinite periodic structure and imposed Floquet periodic boundary conditions to the unit cell (see Figure S18, Supporting Information). Note that the NBR substrate was assumed to have a dielectric constant of 3.4, a loss tangent of 0.2, and a Poisson's ratio of 0.4.^[^ ^84^, ^85^ ^]^ When the MMA was stretched horizontally along the pattern, *P*_h_ and *L*_h_ were increased, while *P*_v_, *L*_v_, and substrate thickness (*t*) were decreased in proportion to the Poisson's ratio of NBR (see the MMA unit cell design in Figure 4a). For simulating EM wave transmission and reception, the numeric ports were added along either side of the stretchable MMA with each distance of 0.8*c*/*λ*, where *c* is the light speed in vacuum, such that the plane wave is excited at one of the ports (the wave excitation was set to ‘on') while the other port acts as a listener port (set to ‘off'). The electric field of the excited wave was aligned parallel to the pattern. The EM wave module simulated the wave propagation by solving the equation derived from Maxwell's equation:

(1) $$\nabla \times \frac{1}{\mu_{r}}\left(\nabla \times \vec{E}\right)-k_{o}^{2}\left(\varepsilon_{r}-\frac{j\sigma }{w\varepsilon_{o}}\right)\vec{E}=0$$

where $\vec{E}$ is the electric field, *µ*_r_ is the relative permeability, *ɛ*_r_ is the relative permittivity, *σ* is the electrical conductivity, *k*_o_ is the wavenumber in the vacuum, and *ɛ*_o_ is the permittivity in the vacuum. For the conductive pattern, the boundary condition was applied as follows,

(2) $$\hat{n}\times \vec{E}=0$$

where $\hat{n}$ is the unit normal vector to the conductor's surface, such that the tangential component of the electric field is zero. For the sake of simplicity, the Floquet periodicity was applied to the periodic boundaries of the unit cell, as expressed by the following Equations (3) and (4):

(3) $${\vec{E}}_{\mathrm{dst}}={\vec{E}}_{\mathrm{src}}e^{-j{\vec{k}}_{f}\cdot \left({\vec{r}}_{\mathrm{dst}}-{\vec{r}}_{\mathrm{src}}\right)}$$

(4) $${\vec{H}}_{\mathrm{dst}}={\vec{H}}_{\mathrm{src}}e^{-j{\vec{k}}_{f}\cdot \left({\vec{r}}_{\mathrm{dst}}-{\vec{r}}_{\mathrm{src}}\right)}$$

where ${\vec{k}}_{f}$ is the wave vector for Floquet periodicity, ${\vec{r}}_{\mathrm{dst}}$ is the position vector of destination, ${\vec{r}}_{\mathrm{src}}$ is the position vector of source, ${\vec{E}}_{\mathrm{dst}}$ is the electric field at destination, ${\vec{E}}_{\mathrm{src}}$ is the electric field at source, ${\vec{H}}_{\mathrm{dst}}$ is the magnetic field at destination, ${\vec{H}}_{\mathrm{src}}$ is the magnetic field at source. In periodic boundary conditions with Floquet periodicity, the source and destination refer to the paired boundaries of the unit cell. The source boundary was the reference boundary where electromagnetic fields are computed, while the destination boundary was the opposite boundary where these fields were mapped. The mapping ensured periodicity by making the fields at the destination boundary phase‐shifted versions of the fields at the source boundary. The phase shift was determined by the Floquet wave vector ${\vec{k}}_{f}$ and the spatial offset ${\vec{r}}_{\mathrm{dst}}-{\vec{r}}_{\mathrm{src}}$. These relationships were essential for accurately simulating periodic structures like metamaterials, enabling the replication of the unit cell's behavior throughout the simulation domain. For the finite element analysis, a mesh was generated in the computational domain. The region containing the printed conductor, which interacts with electric and magnetic fields, was meshed with free triangular elements with minimum and maximum element sizes of 10 and 200 µm, respectively. The rest of the domain was meshed with free tetrahedral elements. The final mesh configuration consisted of 68,353 domain elements, 6,112 boundary elements, and 472 edge elements.

## Conflict of Interest

The authors declare no conflict of interest.

## Supporting information

Supporting Information

Supplemental Movie 1

Supplemental Movie 2

Supplemental Movie 3

## Data Availability

The data that support the findings of this study are available from the corresponding author upon reasonable request.
