# Supplementary material for: Versatile Liquid Metal Composite Inks for Printable, Durable, and Ultra‐Stretchable Electronics
Source: Small. 2025 Jun 11;21(41):2501829. doi: 10.1002/smll.202501829 (PMC12530020; doi:10.1002/smll.202501829)
Supplement: Supplementary file 1 — Supporting Information [file SMLL-21-2501829-s003.pdf]

NANO · MICRO
small

Supporting Information

for *Small*, DOI 10.1002/smll.202501829

Versatile Liquid Metal Composite Inks for Printable, Durable, and Ultra-Stretchable Electronics

Jeongsu Pyeon, Hyeonseung Lee, Wonho Choe, Sanghoo Park and Hyoungsoo Kim**

Versatile Liquid Metal Composite Inks for Printable, Durable, and Ultra-Stretchable Electronics

Jeongsu Pyeon Hyeonseung Lee Wonho Choe Sanghoo Park Hyoungsoo Kim**

J. Pyeon, H Kim
Department of Mechanical Engineering, Korea Advanced Institute of Science and Technology, Daejeon 34141, Republic of Korea
Email: hshk@kaist.ac.kr
H. Lee, W. Choe, S. Park
Department of Nuclear and Quantum Engineering, Korea Advanced Institute of Science and Technology, Daejeon 34141, Republic of Korea
Email: sanghoopark@kaist.ac.kr

Keywords: *Liquid metal, Printing, Self-sintering, Stretchability, Durability, Metamaterial absorber*

	**Materials**	**Conductivity ($S\ m^{-1}$)**	**Stretchability [Max. strain (%)]**
	Galinstan	3.46×10^6[1]	High (> 1000)
**1D Conductive Materials**	Silver (Ag)	6.30×10^7[2]	Low (≤ 70 for AgNW)[3]
	Copper (Cu)	5.96×10^7[2]	Low (≤ 100 for CuNW)[4]
	Carbon Nanotubes (CNTs)	$10^6 - 10^7$[5]	Low (≤ 100)[6]
**2D Conductive Materials**	Graphene	6×10^8[5, 7]	Low (≤ 120)[8]
	Mxene	$10^4 - 10^6$[9]	Relatively high (≤ 300)[10]
**Conductive Polymers**[11]	Polypyrrole (PPy)	$10^2 - 5 \times 10^3$	Low (≤ 80)[12, 13]
	Polyaniline (PANi)	$10^1 - 10^7$	Low (≤ 20)[14]
	Polythiophene (PTh)	$10^{-2} - 10^{-5}$	Low (≤ 10)[15]
	Poly (3,4-ethylene dioxythiophene) (PEDOT)	$3 \times 10^4 - 5 \times 10^4$	Low (≤ 40)[16]

Table S1: Comparative analysis of conductivity and stretchability in various electrode materials

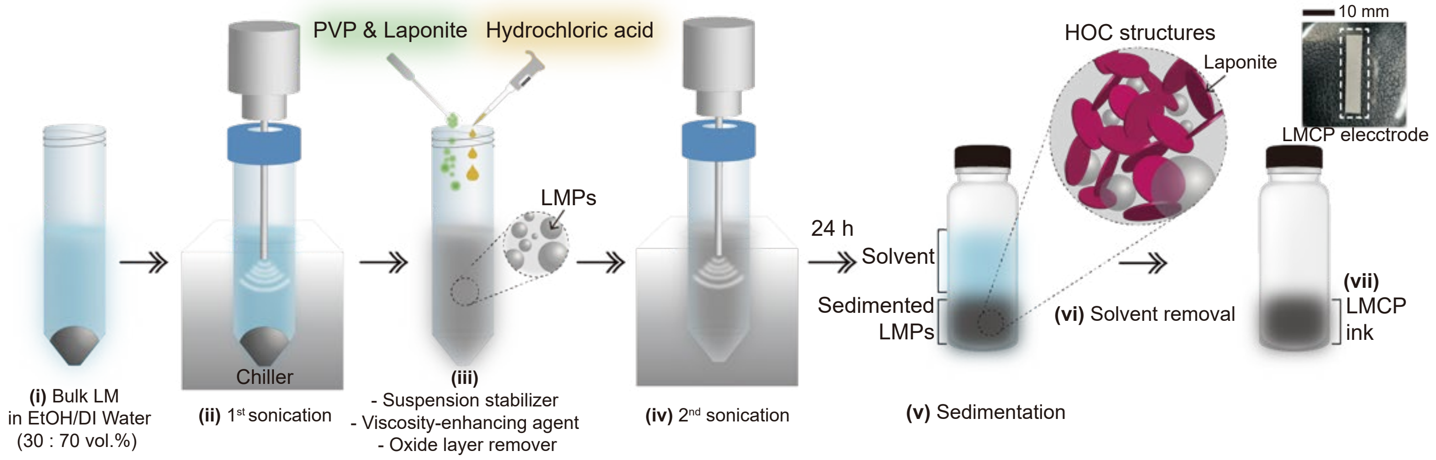

Figure S1. The manufacturing process of LMCP ink. LMCP coating solutions were prepared through the following six steps: (i) bulk LM was added to a mixture of EtOH and DI water, (ii) The mixture solution was treated with ultrasonication, (iii) a liquid metal particle (LMP) dispersion stabilizer (PVP), a solution viscosity-enhancing agent, and an oxide layer remover (HCl) were added, (iv) the mixture underwent ultrasonication once again, (v) the LMP was allowed to naturally settle for 24 hours to separate from the solvents using the difference in density between the LMPs and the solvents. (vii) The LMCP ink fabrication process was finalized by removing the solvent from the top.

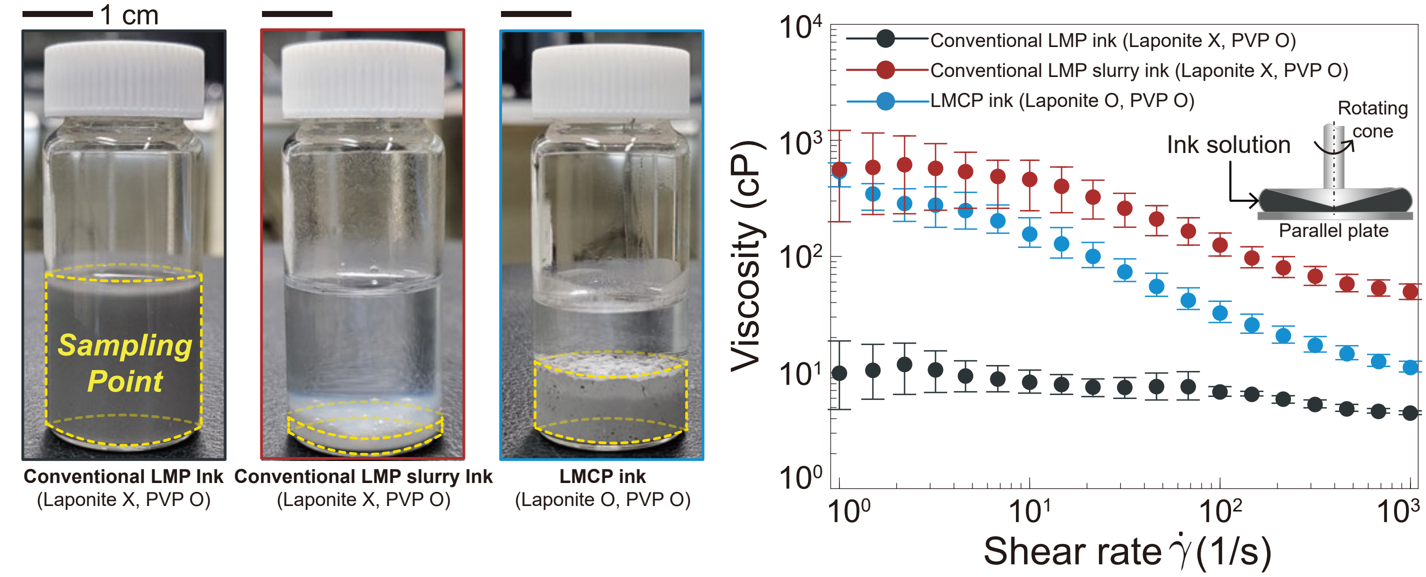

Figure S2. Comparison of viscosity among conventional LMP ink, conventional LMP slurry ink, and LMCP ink. The conventional LMP ink (indicated by black color) and conventional LMP slurry ink (indicated by reddish color) were prepared depending on whether natural sedimentation preceded for 24 hours or not. In both cases, 20 mg of PVPs was added to the LM solution without Laponite. Only for LMCP inks (indicated by bluish color), both 20 mg of PVP and 100 mg of Laponite were added. All samples were collected from the sampling point, marked by the yellow-dashed area, and their viscosities were measured at least three times. Details of the viscosity measurement procedure are described in the Experimental Section. The scale bars are 1 cm.

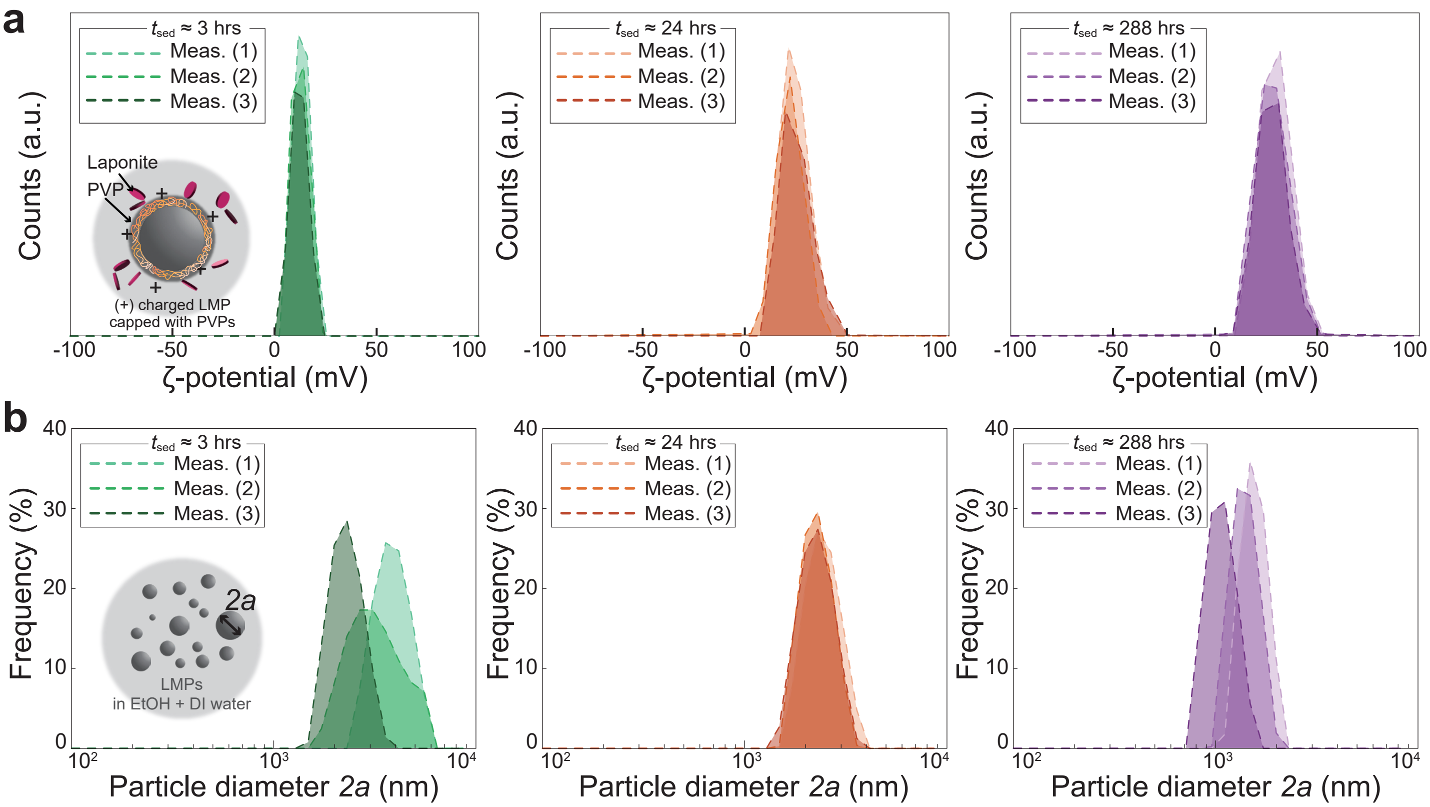

Figure S3. a) ζ-potential and b) particle size distribution of LMCP ink depending on the sedimentation time (t_{sed}).

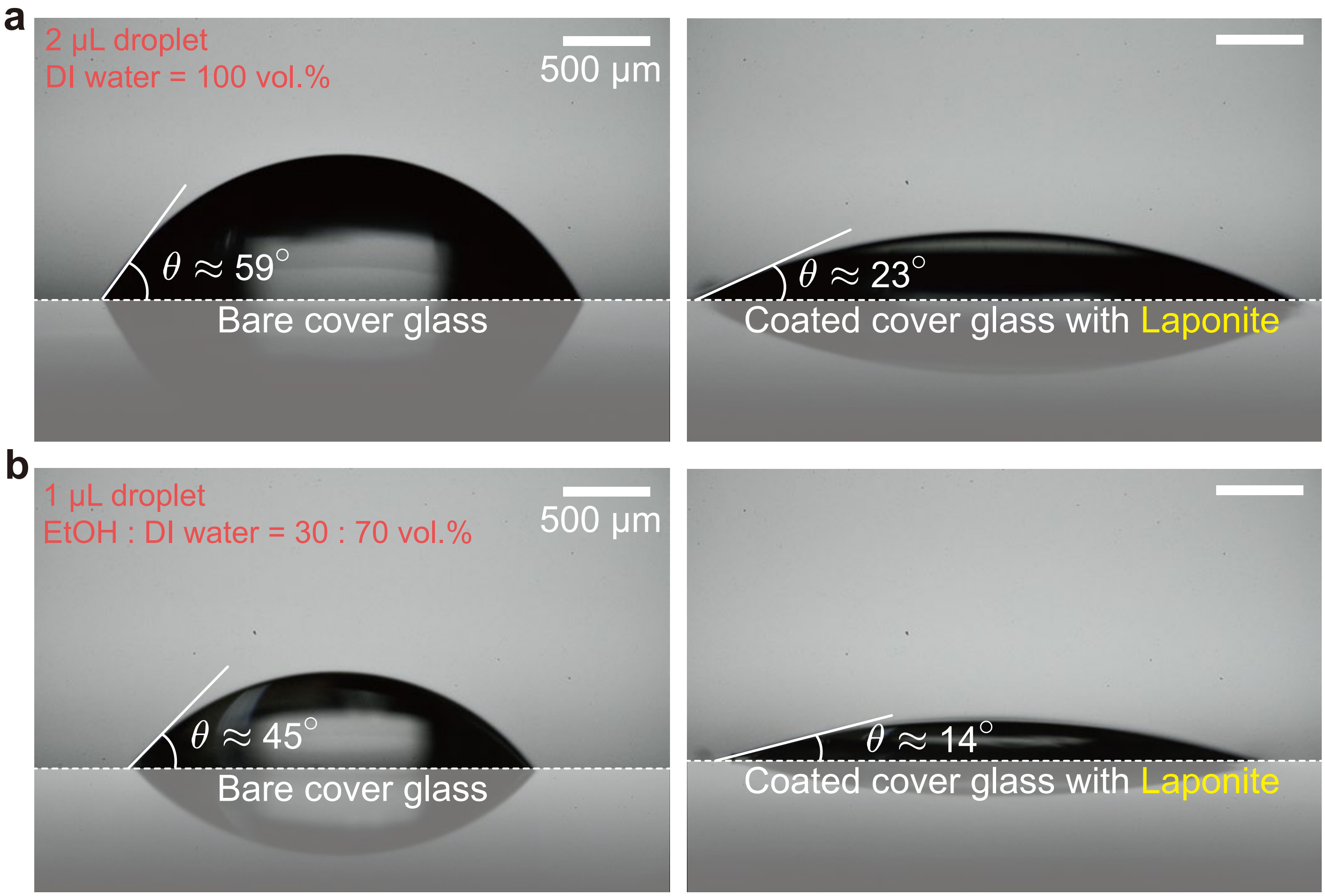

Figure S4. Contact angle measurement results on a surface coated with Laponite. The contact angles of a) pure DI-water droplets and b) EtOH/DI-water mixture (30 : 70 vol.%) droplets were measured and compared on a bare glass substrate and a glass substrate coated with Laponite, respectively. The laponite-coated glass was prepared by dropping a DI water solution containing 2.0 wt% Laponite onto the glass substrate and then evaporating it under ambient conditions.

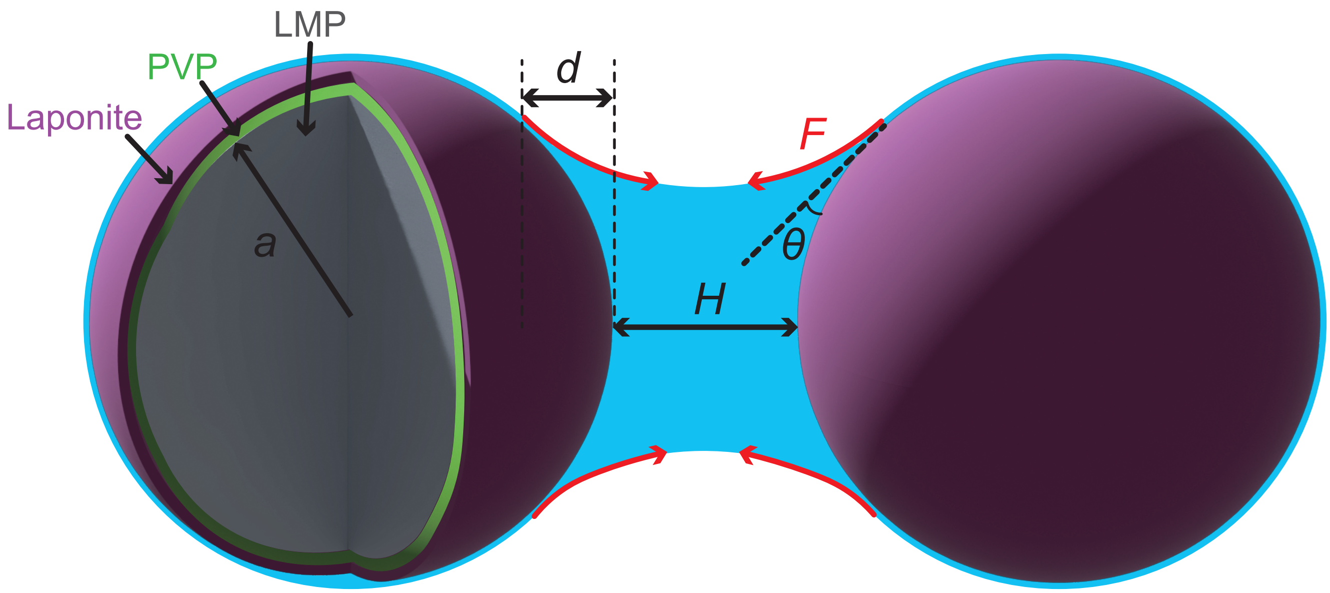

Figure S5. Schematic of the capillary forces between two LMCPs. Here, we assumed that the LMCP consists of a shell structure with three sequential layers: Laponite (purple), PVP (green), and LMP (gray). In this case, the capillary force F can be expressed as $F = 2\pi a\gamma\cos\theta/(1 + H/2d)$, where a is the particle radius (≈ 1 μm), γ is the surface tension (≈ 72 mN m^{-1} for water), θ is the contact angle (≈ 60°), H is the gap distance between LMCPs (≈ 10×10^{-9} m), and d is the immersion length (≈ 2×10^{-11} m) defined by $d = -H + \sqrt{H^2 + V/(\pi a)}$, with V denoting the liquid volume (≈ 1×10^3 nm^3) [17]. The resulting calculation is $F \approx 0.72$ nN.

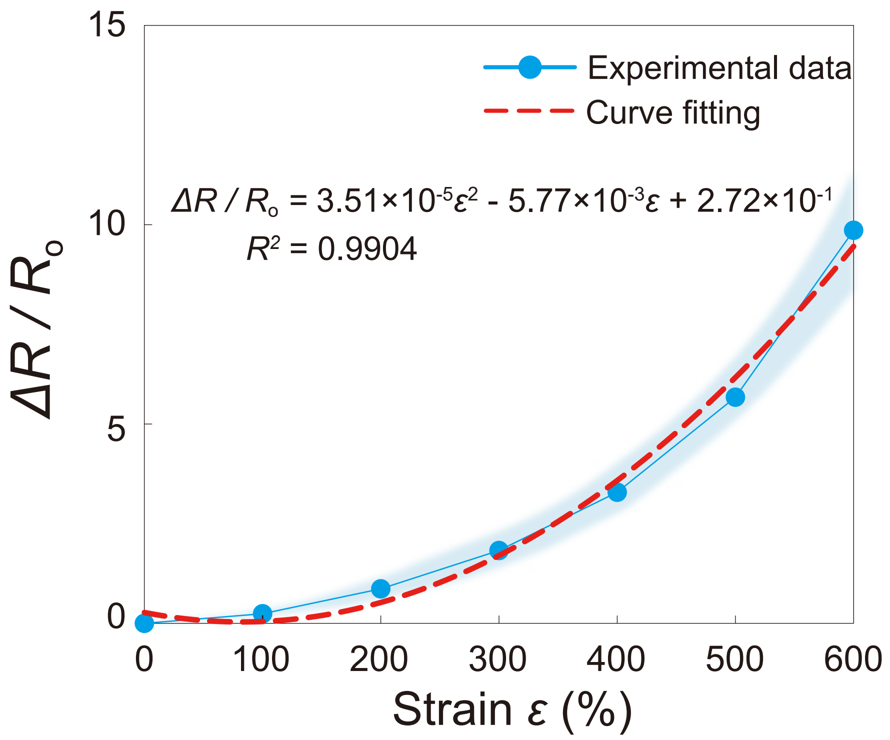

Figure S6. Increasing resistance trend of LMCP electrodes on an elongated WPU substrate. The blue shaded area indicates the margin of experimental error.

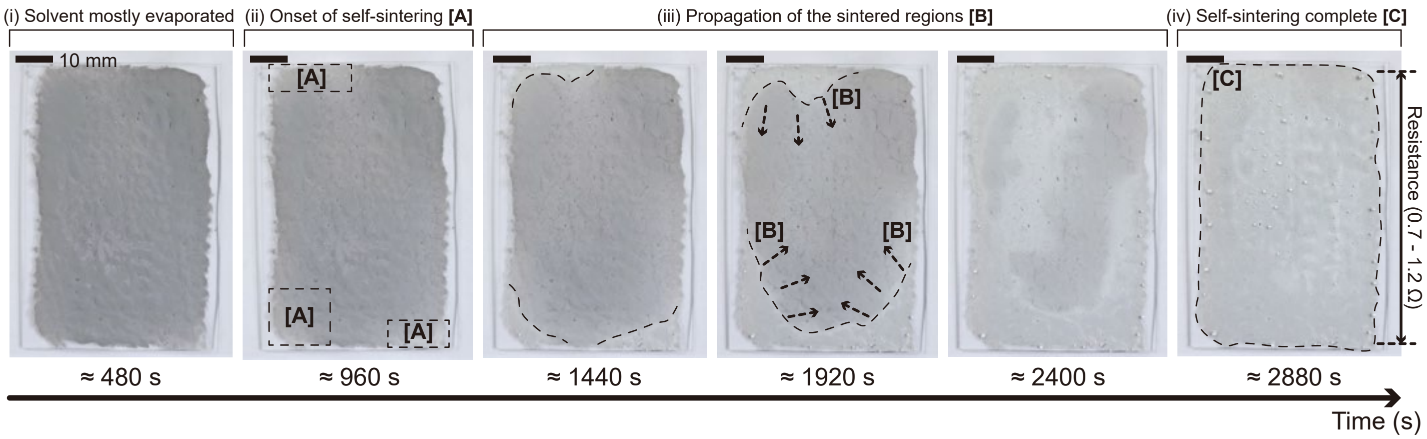

Figure S7. Real-time image sequence of the LMCP electrode sintering process. LMCP ink was printed and dried on a glass substrate. The bright white region indicates the sintered area, which gradually propagates over time as the remaining solvent evaporates. The two-point resistance measured between the farthest ends of the large-area rectangular electrode was approximately 0.7–1.0 Ω.

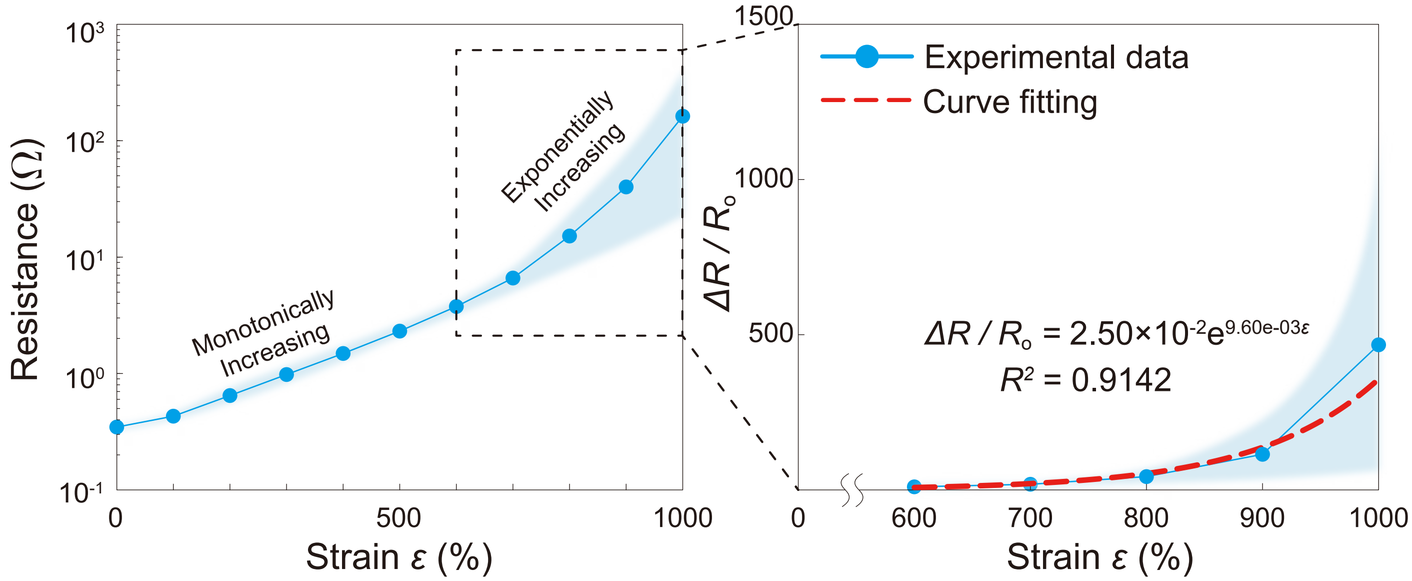

Figure S8. Analysis of LMCP electrode resistance increase trend under ultra-stretching conditions. The blue-shaded area illustrates the experimental error margins.

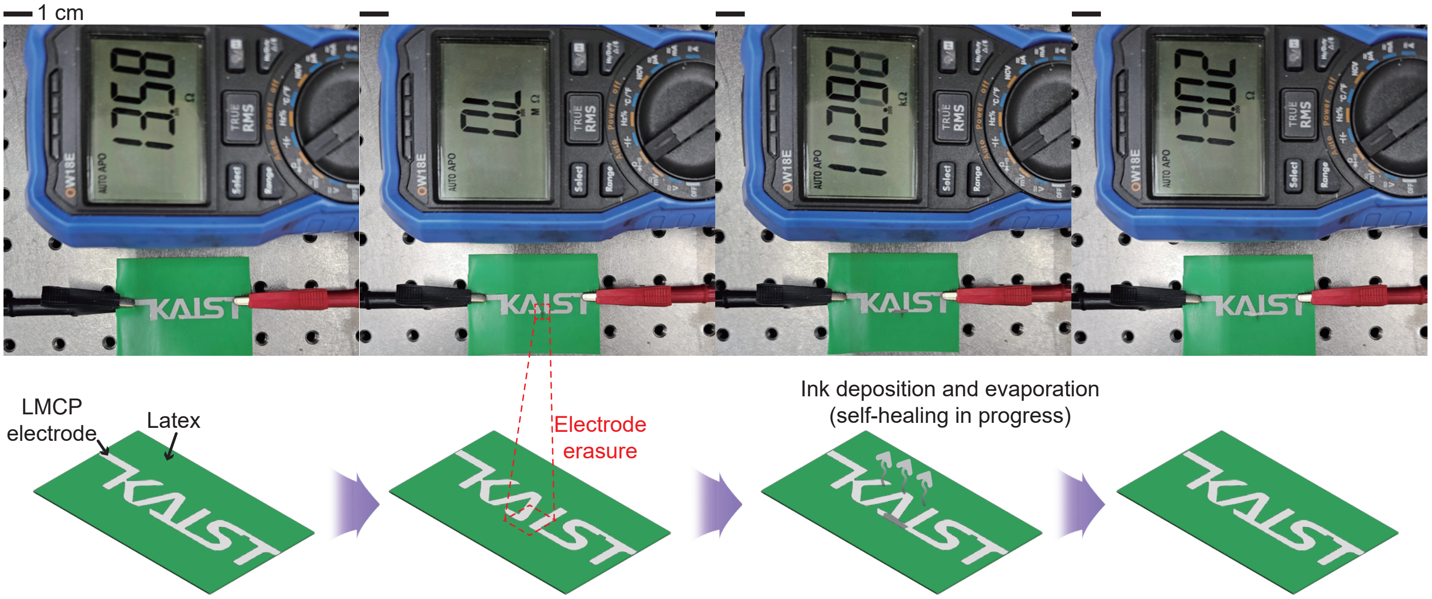

Figure S9. Self-healing properties of LMCP electrodes on a stretchable and flexible substrate. To cut off the LMCP conduction path, the electrode was partially erased using an isopropyl alcohol (IPA) solution. The ink was then reapplied to the disconnected area and left to evaporate, successfully restoring the electrode's electrical conductivity.

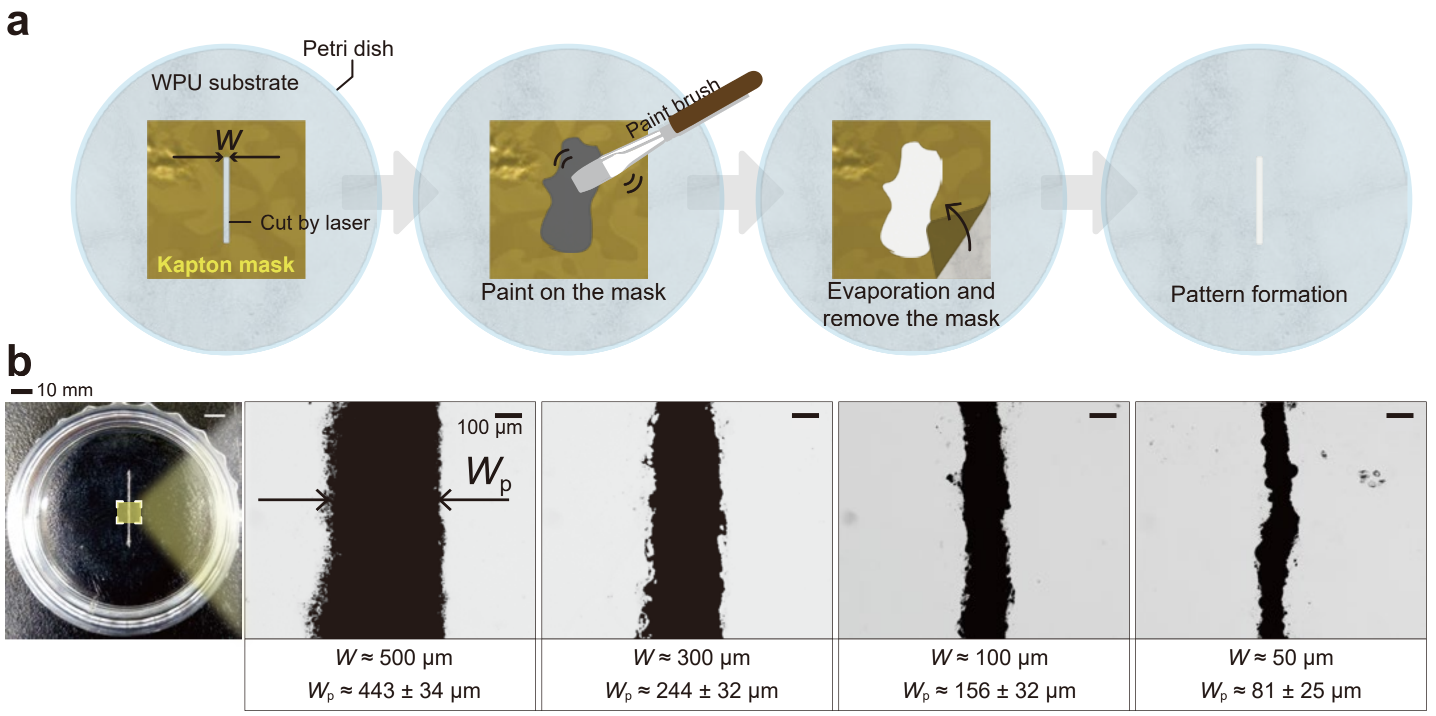

Figure S10. Resolution test results of LMCP ink using stencil printing. a) Schematic of the stencil printing procedure. b) Resulting patterns obtained from (a). W is the laser-cut mask width, and W_p is the printed electrode width.

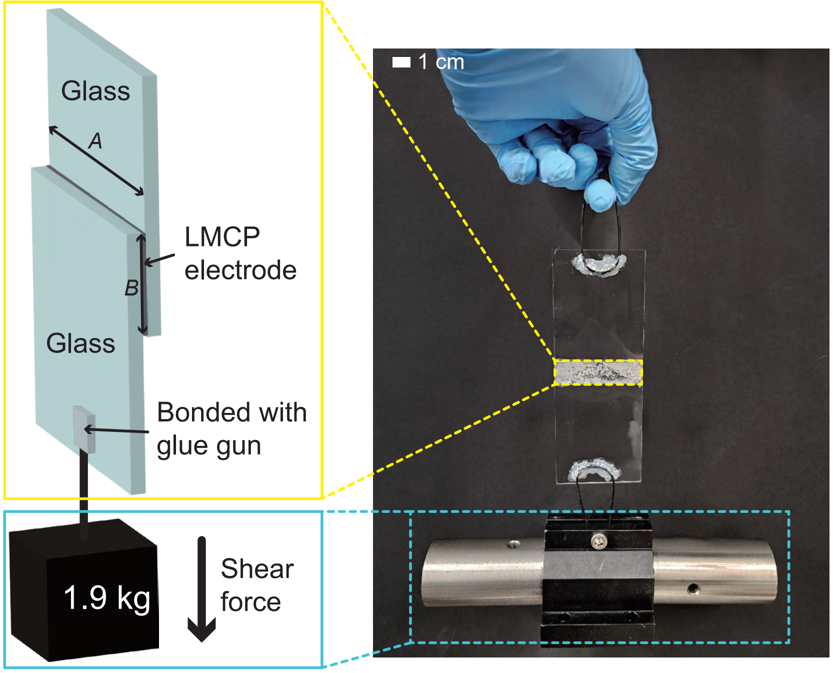

Figure S11. Lap shear test for adhesive strength of LMCP electrodes. The LMCP electrode was printed on a 5.0 cm × 1.3 cm (glass width A × printing length B) overlapping area between the glass substrates, with a downward shear force of approximately 18.6 N applied. The adhesive strength was calculated by dividing the applied shear force by the overlapping area.

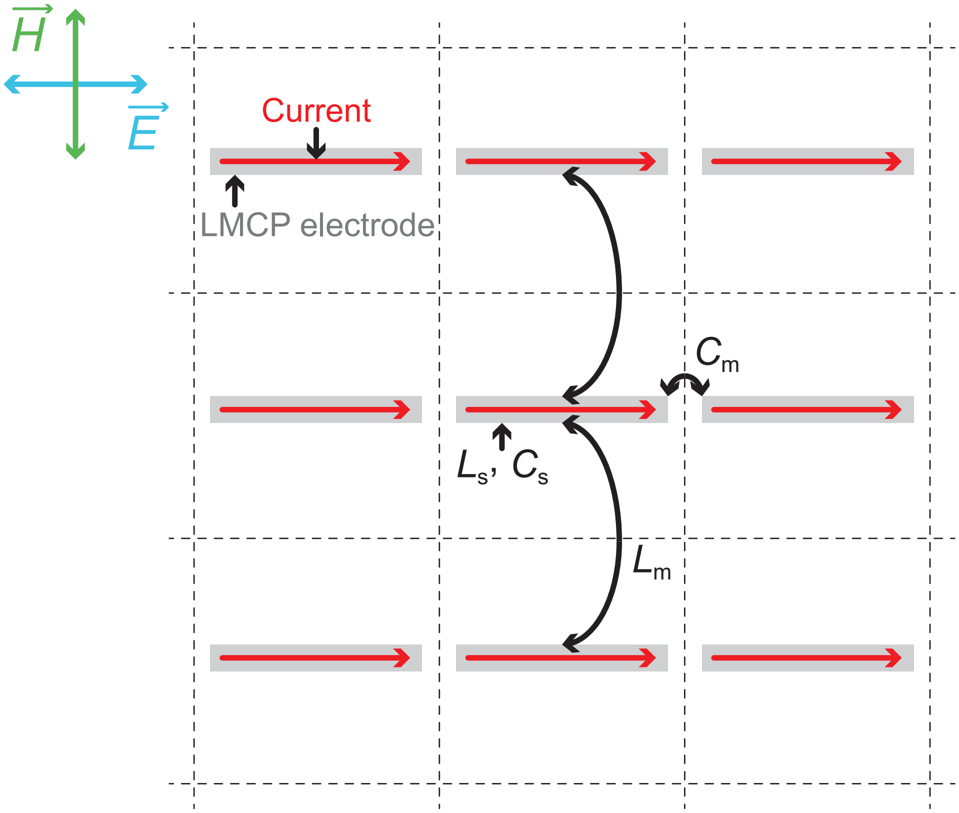

Figure S12. Simplified equivalent circuit models for LCMP-based MMAs. C_s is the self-capacitance, C_m is the mutual capacitance, C_t is the total capacitance, L_s is the self-inductance, L_m is the mutual inductance, and L_t is the total inductance.

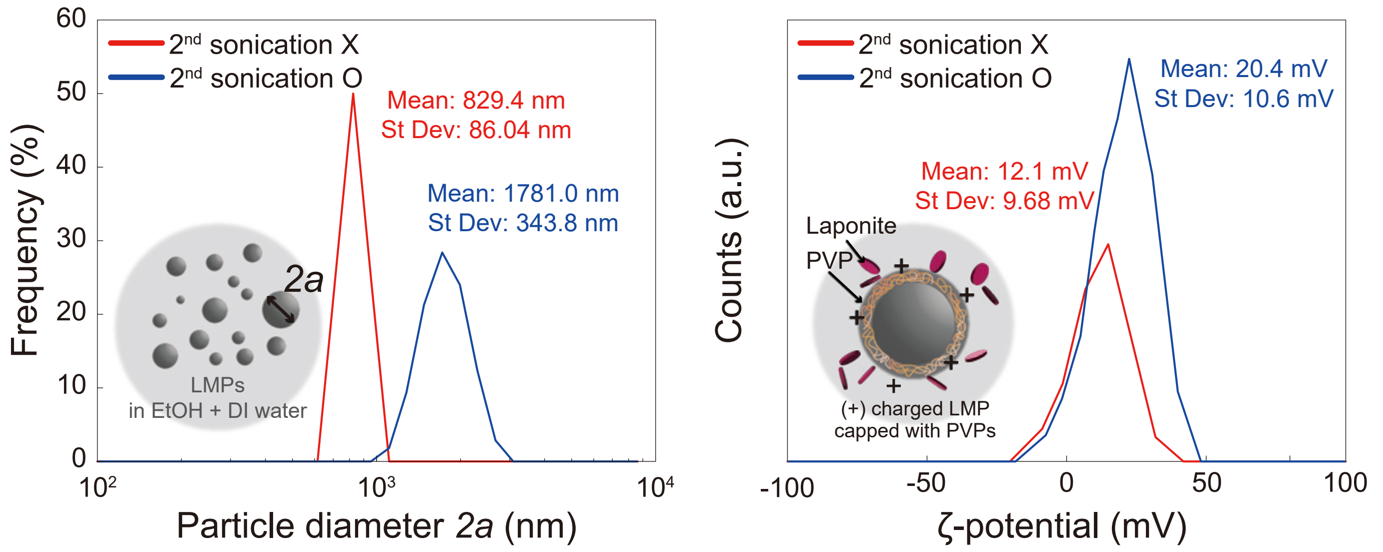

Figure S13. Comparison of particle size distribution and ζ-potential with/without second sonication. The (v–vii) processes in Figure S1 were not performed when preparing the inks. Measurements were repeated at least three times.

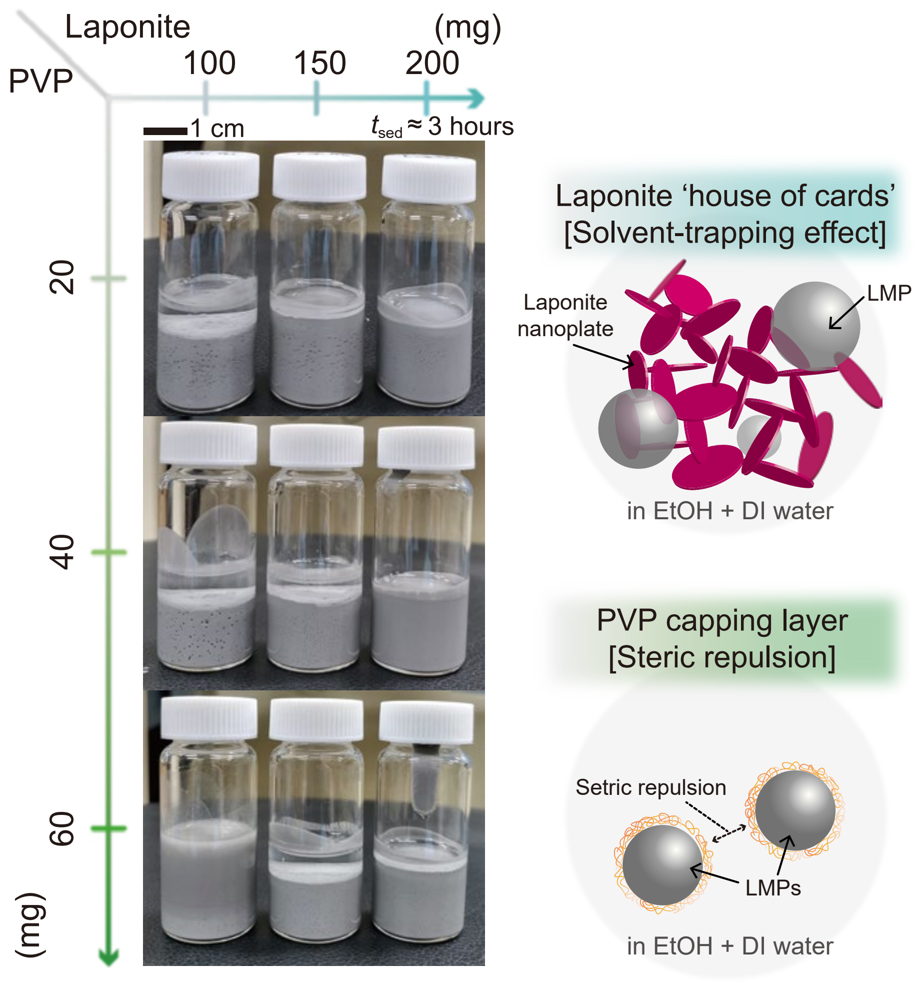

Figure S14. LMCP ink composition optimization process. The natural sedimentation behaviors of LMCP inks were examined under varying concentrations of PVP and Laponite. During sedimentation, Laponite nanoplates formed 'house-of-cards' (HOC) structures, trapping the surrounding liquid solvent within their self-assembled structures. Concurrently, PVP agents adhered to the surface of the LMP, inducing steric repulsion among them.

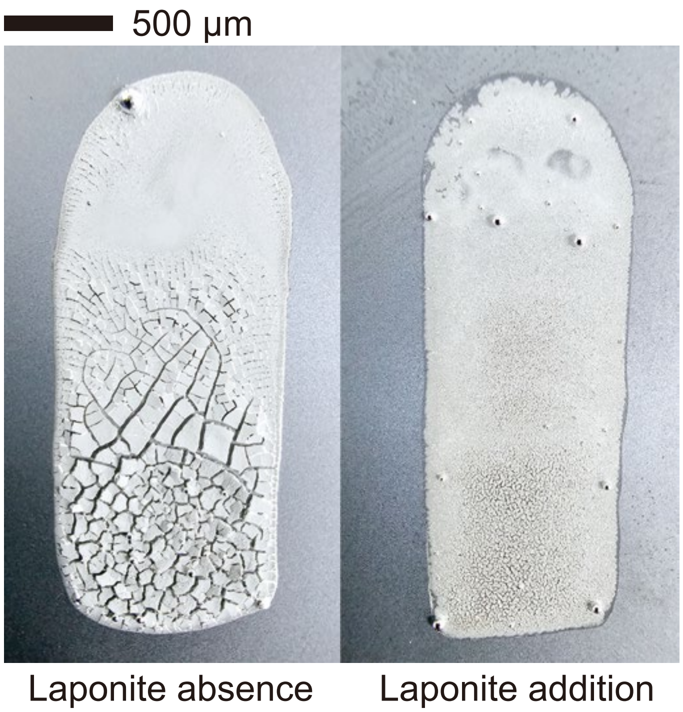

Figure S15. Comparison of dried electrode patterns, without (left) and with (right) Laponite additions. All electrodes were fabricated using a one-touch brushing on silicon wafers.

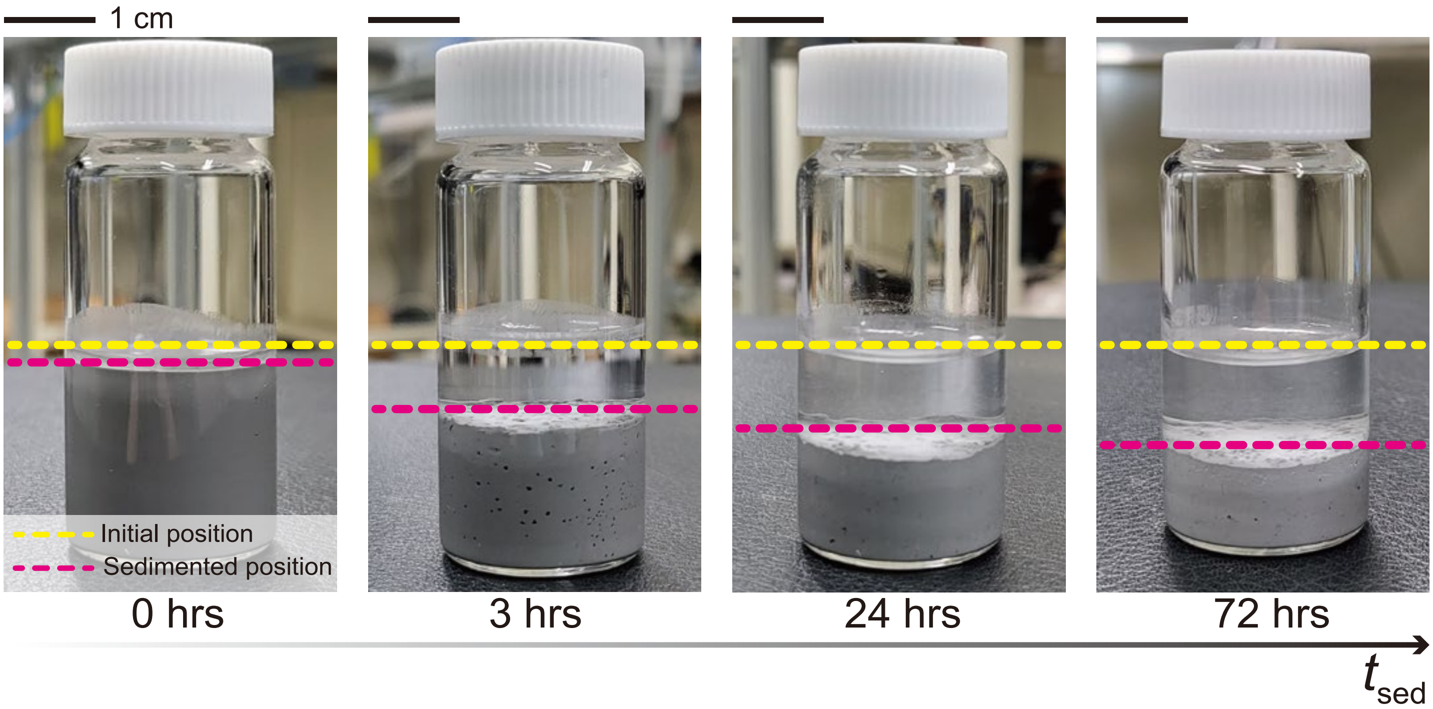

Figure S16. Time-dependent phase separation of the LMCP inks. Sequential side-view images of the LMCP ink were captured depending on the different sedimentation time t_{sed}.

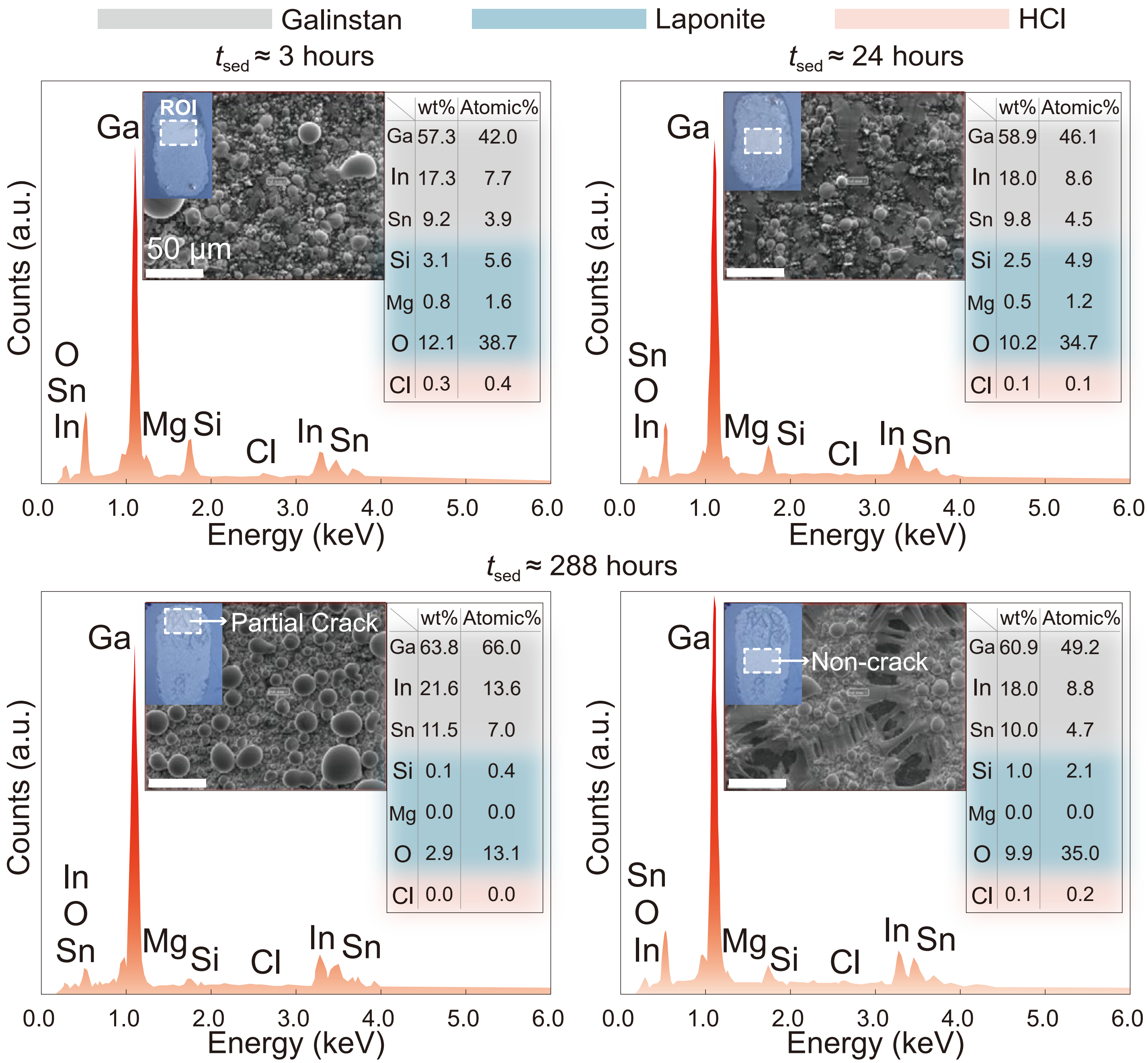

Figure S17. Optimization for the natural sedimentation time (t_{sed}). Energy-Dispersive X-ray Spectroscopy (EDS) was conducted at different sedimentation times ($t_{sed} \approx 3$, 24, and 288 hours) and under conditions both with and without cracks. The uncertainty of EDS analysis is approximately 9.36 ± 0.99 %. All white scale bars represent 50 µm. Each DLS measurement was performed at least three times.

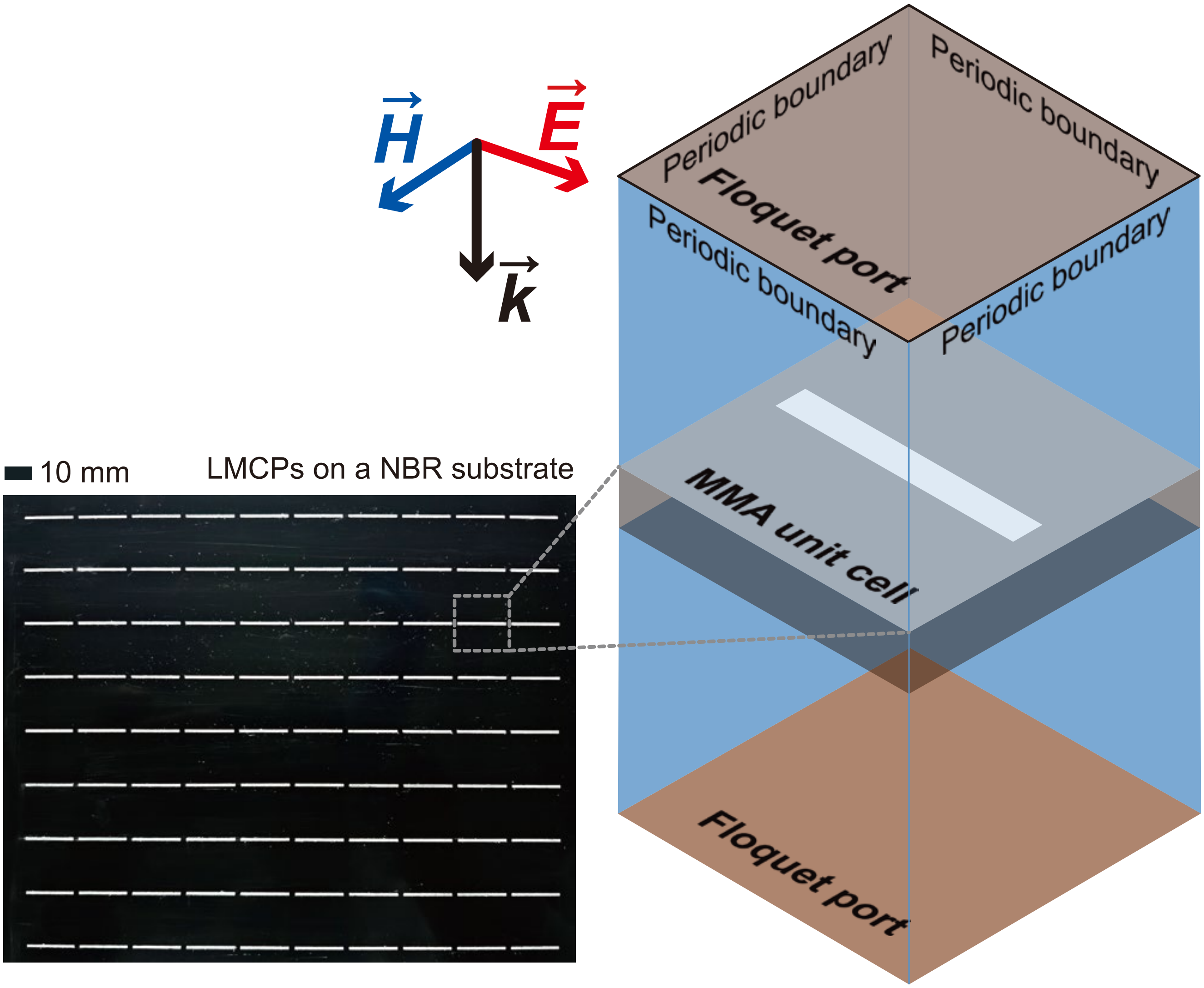

Figure S18. Electromagnetic (EM) simulation model of the stretchable MMA. The blue region represents the periodic boundary, and the orange region indicates the Floquet port used to generate and receive EM waves.

Supporting Note 1. Estimation of the Minimum Amount of HCl Required to Completely Remove the Oxide Layers on Liquid Metal Particle Surfaces

To clarify the rationale behind our use of 50 µL HCl in the ink formulation, we performed a theoretical estimation of the minimum amount of HCl required to completely remove the Ga_2O_3 oxide layers from the surface of LM particles dispersed in the ethanol–water mixture.

Assumptions:

- Volume of Galinstan: 0.5 mL
- Average LM particle diameter: 2.2 ± 0.5 µm
- Oxide layer thickness: 3 nm [18]
- Density of Ga_2O_3: 5.88 g cm^{-1} [19]
- Molar mass of Ga_2O_3: 187.44 g mol^{-1}
- Reaction: $Ga_2O_3 + 6H^+ \rightarrow 2Ga^{3+} + 3H_2O$
- HCl concentration: 12 mol L^{-1} (from 37 wt% HCl)

Calculation:

$$\text{Radius of LM particle: } r = 1.1 \times 10^{-4}\,\text{cm}$$

$$\text{Number of particles: } N = \frac{0.5\,\text{cm}^3}{\frac{4}{3}\pi r^3} \approx 8.97 \times 10^{10}$$

$$\begin{aligned} \text{Total oxide volume: } V_{\text{oxide}} &= N \cdot 4\pi r^2 t \\ &= 8.97 \times 10^{10} \cdot 4\pi (1.1 \times 10^{-4}\,\text{cm})^2 \cdot 3 \times 10^{-7}\,\text{cm} \\ &\approx 4.09 \times 10^{-3}\,\text{cm}^3 \end{aligned}$$

$$\text{Mass of Ga}_2\text{O}_3 = \rho \cdot V = 5.88\,\text{g/cm}^3 \cdot 4.09 \times 10^{-3}\,\text{cm}^3 \approx 2.41 \times 10^{-2}\,\text{g}$$

$$\text{Moles of Ga}_2\text{O}_3 = \frac{2.41 \times 10^{-2}\,\text{g}}{187.44\,\text{g/mol}} \approx 1.28 \times 10^{-4}\,\text{mol}$$

$$\text{Required moles of H}^+ = 6 \cdot 1.28 \times 10^{-4}\,\text{mol} = 7.70 \times 10^{-4}\,\text{mol}$$

$$\text{Required volume of HCl} = \frac{7.70 \times 10^{-4}\,\text{mol}}{12\,\text{mol/L}} \approx \boxed{64.17\,\mu\text{L}}$$

This result shows that the theoretical minimum HCl volume for complete oxide removal is approximately 64 µL, which is closely comparable to the 50 µL of HCl used in our optimized formulation. This small difference likely arises from uncertainties in the underlying assumptions—such as the idealization of perfectly spherical LM particles, potential measurement errors in the DLS analysis, a uniform particle size distribution, a fixed 3 nm oxide shell thickness, and inaccuracies in the reported properties of Ga_2O_3 due to its complex chemical behavior.

References

[1] S. Cheng, Z. Wu, *Lab on a Chip* **2012**, *12*, 16 2782.

[2] Y. Lin, J. Genzer, M. D. Dickey, *Advanced Science* **2020**, *7*, 12 2000192.

[3] Y. Chen, R. S. Carmichael, T. B. Carmichael, *ACS Applied Materials & Interfaces* **2019**, *11*, 34 31210.

[4] P. Tran, N.-H. Tran, J.-H. Lee, *Scientific Reports* **2022**, *12*, 1 8967.

[5] Y. Wang, G. J. Weng, *Micromechanics and Nanomechanics of Composite Solids* **2018**, 123–156.

[6] S. Hong, J. Lee, K. Do, M. Lee, J. H. Kim, S. Lee, D.-H. Kim, *Advanced Functional Materials* **2017**, *27*, 48 1704353.

[7] M. H. Fahmy Taha, H. Ashraf, W. Caesarendra, *Applied System Innovation* **2020**, *3*, 3 32.

[8] N. Liu, A. Chortos, T. Lei, L. Jin, T. R. Kim, W.-G. Bae, C. Zhu, S. Wang, R. Pfattner, X. Chen, R. Sinclair, Z. Bao, *Science Advances* **2017**, *3*, 9 e1700159.

[9] C. Ma, M.-G. Ma, C. Si, X.-X. Ji, P. Wan, *Advanced Functional Materials* **2021**, *31*, 22 2009524.

[10] Y. Zhou, K. Maleski, B. Anasori, J. O. Thostenson, Y. Pang, Y. Feng, K. Zeng, C. B. Parker, S. Zauscher, Y. Gogotsi, J. T. Glass, C. Cao, *ACS Nano* **2020**, *14*, 3 3576.

[11] X. Chen, B. Li, Y. Qiao, Z. Lu, *Micromachines* **2019**, *10*, 11 788.

[12] C.-W. Tsao, X.-C. Guo, W.-W. Hu, *RSC Advances* **2016**, *6*, 114 113344.

[13] W. Muhammad, S.-D. Kim, *Polymers* **2023**, *15*, 7 1714.

[14] Z. Huang, Z. Ji, Y. Feng, P. Wang, Y. Huang, *Polymer International* **2021**, *70*, 4 437.

[15] Q. Chen, X. Wang, F. Chen, N. Zhang, M. Ma, *Chemical Engineering Journal* **2019**, *368* 933.

[16] J. H. Lee, Y. R. Jeong, G. Lee, S. W. Jin, Y. H. Lee, S. Y. Hong, H. Park, J. W. Kim, S.-S. Lee, J. S. Ha, *ACS Applied Materials & Interfaces* **2018**, *10*, 33 28027.

[17] Y. I. Rabinovich, M. S. Esayanur, B. M. Moudgil, *Langmuir* **2005**, *21*, 24 10992.

[18] I. D. Joshipura, K. A. Persson, V. K. Truong, J.-H. Oh, M. Kong, M. H. Vong, C. Ni, M. Alsafatwi, D. P. Parekh, H. Zhao, *Langmuir* **2021**, *37*, 37 10914.

[19] J. Jesenovec, M. H. Weber, C. Pansegrau, M. D. McCluskey, K. G. Lynn, J. S. McCloy, *Journal of Applied Physics* **2021**, *129*, 24.
